# Supplementary material for: Genome-wide meta-analysis of muscle weakness identifies 15 susceptibility loci in older men and women
Source: Nat Commun. 2021 Jan 28;12:654. doi: 10.1038/s41467-021-20918-w (PMC7844411; doi:10.1038/s41467-021-20918-w)
Supplement: Supplementary file 1 — Supplementary Information [file 41467_2021_20918_MOESM1_ESM.pdf]

Genome-wide meta-analysis of muscle weakness identifies 15 susceptibility loci in older men and women

G. Jones *et al.*

Supplementary Information

Supplementary Note 1 3
Supplementary Methods 5
Cohorts 5
Atherosclerosis Risk in Communities (ARIC) study 5
Berlin Aging Study II (BASE-II) 6
B-Vitamins for the PRevention Of Osteoporotic Fractures (BPROOF) study 6
Cardiovascular Health Study (CHS) 6
European Prospective Investigation of Cancer (EPIC) Norfolk 7
Framingham Heart Study 8
Health and Retirement Study (HRS) 8
InCHIANTI study 9
LASA: Longitudinal Aging Study Amsterdam 9
Long-Life Family Study 10
MrOS (Osteoporotic Fractures in Men) study 12
ROSMAP: the Religious Orders Study (ROS) and Memory and Aging Project (MAP) 13
Rotterdam Study 13
Study of Health in Pomerania (SHIP) 13
Toledo Study for Healthy Aging (TSHA) 14
UK Biobank (UKB) 15
Wisconsin Longitudinal Study (WLS) 15
GWAS meta-analysis methods 16
Supplementary Figure 1: QQ plot of EWGSOP low grip GWAS results 17
Supplementary Figures 2A-O: LocusZoom plots for the 15 EWGSOP low grip loci 18
Supplementary Figure 2A: rs34415150 (chr6:32560477) 18
Supplementary Figure 2B: rs143384 (chr20:34025756) 19
Supplementary Figure 2C: rs62102286 (chr18:46592408) 19
Supplementary Figure 2D: rs3118903 (chr13:51099577) 20
Supplementary Figure 2E: rs13107325 (chr4:103188709) 20
Supplementary Figure 2F: rs11236213 (chr11:74394369) 21
Supplementary Figure 2G: rs34464763 (chr12:15032860) 21

Supplementary Figure 2H: rs143459567 (chr16:24600412) 22
Supplementary Figure 2I: rs2899611 (chr15:58327347) 22
Supplementary Figure 2J: rs958685 (chr2:70703847) 23
Supplementary Figure 2K: rs7624084 (chr3:141093285) 23
Supplementary Figure 2L: rs79723785 (chr19:55818225) 24
Supplementary Figure 2M: rs10952289 (chr7:150524681) 24
Supplementary Figure 2N: rs8061064 (chr16:53912364) 25
Supplementary Figure 2O: rs12140813 (chr1:227776827) 25
Supplementary Figure 3: Plot of Mendelian randomisation analysis of depression SNPs on low grip strength (EWGSOP) in females only 26
Supplementary Table 1: Summary of cohorts included in the meta-analysis 27
Supplementary Table 2: Genomic risk loci associated with low grip strength EWGSOP definition, Female only 28
Supplementary Table 3: Genomic risk loci associated with low grip strength EWGSOP definition, Male only 29
Supplementary Table 4: Genomic risk loci associated with low grip strength FNIH definition 30
Supplementary Table 5: Genetic correlations with low grip strength (EWGSOP) 31
Supplementary Table 6: ICD-10 and UK Biobank self-reported codes used in sensitivity analysis 32
Supplementary References 33

Supplementary Note 1

GJ, DM and LCP are supported by the University of Exeter Medical School and the University of Connecticut School of Medicine. This work was generously supported by an award to DM by the UK Medical Research Council (grant number MR/M023095/1). CLK was supported by an IPA agreement (#20170526) with LF. Access to UK Biobank Resource was granted under Application Number 14631. We would like to thank UK Biobank participants and coordinators for this dataset.

The Atherosclerosis Risk in Communities study has been funded in whole or in part with Federal funds from the National Heart, Lung, and Blood Institute, National Institutes of Health, Department of Health and Human Services (contract numbers HHSN268201700001I, HHSN268201700002I, HHSN268201700003I, HHSN268201700004I and HHSN268201700005I), R01HL087641, R01HL059367 and R01HL086694; National Human Genome Research Institute contract U01HG004402; and National Institutes of Health contract HHSN268200625226C. The authors thank the staff and participants of the ARIC study for their important contributions. Infrastructure was partly supported by Grant Number UL1RR025005, a component of the National Institutes of Health and NIH Roadmap for Medical Research.

Cardiovascular Health Study: This CHS research was supported by NHLBI contracts HHSN268201200036C, HHSN268200800007C, HHSN268201800001C, N01HC55222, N01HC85079, N01HC85080, N01HC85081, N01HC85082, N01HC85083, N01HC85086; and NHLBI grants U01HL080295, R01HL087652, R01HL105756, R01HL103612, R01HL120393, and U01HL130114 with additional contribution from the National Institute of Neurological Disorders and Stroke (NINDS). Additional support was provided through R01AG023629 from the National Institute on Aging (NIA). A full list of principal CHS investigators and institutions can be found at CHS-NHLBI.org. The provision of genotyping data was supported in part by the National Center for Advancing Translational Sciences, CTSI grant UL1TR001881, and the National Institute of Diabetes and Digestive and Kidney Disease Diabetes Research Center (DRC) grant DK063491 to the Southern California Diabetes Endocrinology Research Center. The content is solely the responsibility of the authors and does not necessarily represent the official views of the National Institutes of Health.

We are grateful to all the participants who have been part of the EPIC-Norfolk project and to the many members of the study teams at the University of Cambridge who have enabled this research. The study is funded by Medical Research Council (MR/N003284/1, MC-UU_12015/1, MC_PC_13048) Cancer Research UK (C864/A14136).

The Framingham Heart Study (FHS): The authors thank the Framingham Heart Study participants and staff for their dedication to the study. The Framingham Heart Study phenotype-genotype analyses were supported by NIA R01AG29451 JMM, KLL. The Framingham Heart Study of the National Heart Lung and Blood Institute of the National Institutes of Health and Boston University School of Medicine was supported by the National Heart, Lung and Blood Institute's Framingham Heart Study (Contract No. N01-HC-25195 and HHSN26820150001) and its contract with Affymetrix, Inc for genotyping services (Contract No. N02-HL-6-4278). Analyses reflect intellectual input and resource development from the Framingham Heart Study investigators participating in the SNP Health Association Resource (SHARe) project. The authors are pleased to acknowledge that the computational work reported on in this paper was performed on the Shared Computing Cluster which is administered by Boston University's Research Computing Services. URL: www.bu.edu/tech/support/research/. Dr. Kiel's effort was supported by a grant from the National Institute of Arthritis Musculoskeletal and Skin Diseases (R01 AR41398). This project was additionally supported by National Institute on Aging (AG08122, AG033193, AG016495, U24AG051129).

The Longitudinal Aging Study Amsterdam (LASA) is supported by a grant from the Netherlands Ministry of Health, Welfare and Sport, Directorate of Long-Term Care. Genotyping using the Axiom-NL Array was funded by the EMGO Institute for Health and Care Research. The authors would like to thank all the participants, researchers and supporting staff of the Longitudinal Aging Study Amsterdam.

Long Life Family Study: This work was supported by the United States' National Institutes of Health, National Institute on Aging (U19-AG063893-01, U01-AG023712, U01-AG23744, U01-AG023746, U01-AG023749, and U01-AG023755). AJS was supported by a career development award from the National Institute on Aging (K01 AG057726).

ROSMAP was supported by the following grants: P30AG10161, R01AG15819, R10AG17917, U01AG46152 and U01AG61356.

The Rotterdam Study is supported by the Erasmus MC University Medical Center and Erasmus University Rotterdam; The Netherlands Organisation for Scientific Research (NWO); The Netherlands Organisation for Health Research and Development (ZonMw); the Research Institute for Diseases in the Elderly (RIDE); The Netherlands Genomics Initiative (NGI); the Ministry of Education, Culture and Science; the Ministry of Health, Welfare and Sports; the European Commission (DG XII); and the Municipality of Rotterdam. The contribution of inhabitants, general practitioners and pharmacists of the Ommoord district to the Rotterdam Study is gratefully acknowledged.

SHIP is part of the Community Medicine Research net of the University of Greifswald, Germany, which is funded by the Federal Ministry of Education and Research (grants no. 01ZZ9603, 01ZZ0103, and 01ZZ0403), the Ministry of Cultural Affairs as well as the Social Ministry of the Federal State of Mecklenburg-West Pomerania, and the network 'Greifswald Approach to Individualized Medicine (GANI_MED)' funded by the Federal Ministry of Education and Research (grant 03IS2061A). Genome-wide data have been supported by the Federal Ministry of Education and Research (grant no. 03ZIK012) and a joint grant from Siemens Healthineers, Erlangen, Germany and the Federal State of Mecklenburg- West Pomerania. The University of Greifswald is a member of the Caché Campus program of the InterSystems GmbH.

The Toledo Study for Healthy Aging (TSHA) was funded by grants from the Spanish Ministry of Economy, Industry and Competitiveness, co-financed by the European Regional Development Funds (RD120001/0043), the FIS16/02125 from the Carlos III Institute of Health, the Community of Madrid 2018T1/BMD11226 and the Centro de Investigación Biomédica en Red en Fragilidad y Envejecimiento Saludable—CIBERFES (CB16/10/00464). The genotyping was performed at the Spanish National Cancer Research Centre, in the Human Genotyping lab, a member of CeGen, PRB3 and is supported by grant PT17/0019, of the PE I+D+i 2013-2016, funded by ISCIII and ERDF.Since 1991, The Wisconsin Longitudinal Study (WLS) (of the University of Wisconsin-Madison, has been supported principally by the National Institute on Aging (AG-9775, AG-21079, AG-033285, and AG-041868, R01 AG041868-01A1), with additional support from the Vilas Estate Trust, the National Science Foundation, the Spencer Foundation, and the Graduate School of the University of Wisconsin-Madison. Since 1992, data have been collected by the University of Wisconsin Survey Center. The opinions expressed herein are those of the authors. A public use file of data from the Wisconsin Longitudinal Study is available from the Wisconsin Longitudinal Study, University of Wisconsin-Madison, 1180 Observatory Drive, Madison, Wisconsin 53706 and at http://www.ssc.wisc.edu/WLSresearch/data/.

Supplementary Methods

Cohorts

22 studies with a total of 254,894 participants of European ancestries and age 60 or older were included in the analysis low grip strength. See Supplementary Table 1 for number of cases/controls in each cohort. Here follows individual study descriptions:

Atherosclerosis Risk in Communities (ARIC) study

The Atherosclerosis Risk in Communities (ARIC) Study is a population-based prospective cohort study of cardiovascular disease and includes a total of 15,792 participants aged 45-64 years at baseline (1987-89), chosen by probability sampling from four US communities(1). Cohort members completed five clinic examinations, conducted approximately three years apart between 1987 and 1998, with a fifth visit conducted from 2011 – 2013. Clinic examinations included assessment of cardiovascular risk factors, self-reported medical family history, employment and educational status, diet, physical activity, comorbidity, clinical and laboratory measurements. The present analyses were restricted to participants of European descent and utilized measurements from the Visit 5 grip strength assessment. Grip strength in kilograms of force was assessed using Jamar Hydraulic Hand Dynamometer in the participant's preferred hand (usually the dominant). The better of two trials was used for the dynapenia definitions and subsequent association analyses.

Genotyping was performed using the Affymetrix Genome-Wide Human SNP Array 6.0 (Affymetrix, Santa Clara, CA, USA). Exclusions at the individual level included removing first-degree relatives, ancestry outliers, samples with low call rate (<95%) or unexpected duplicates, and samples with a mismatch between called and phenotypic sex. SNPs were excluded due to having a low call rate for the combined sample (<95%), no chromosome location, being monomorphic in both the European and African descended genotyped individuals, and having an HWE p-value <10-6. Before imputation, additional quality control was completed using the "HRC/1KG Imputation Preparation and Checking Tool" developed by Will Rayner (version 4.2.5). SNPs with incorrect strand or reference/alternate allele designations were fixed accordingly and further SNPs were excluded due to mismatches with the 1000 Genomes reference panel, having allele frequency differences >0.2 from the European 1000 Genomes data, and being palindromic with frequencies >0.4. A total of 752,325 SNPs were submitted for imputation on the Michigan Imputation Server using the Haplotype Reference Consortium (HRC) r1.1 2016 reference panel with the following options chosen: phasing, Eagle v2.3; population, EUR (for quality control purposes); mode, Quality Control & Imputation.

After filtering for age, phenotype, genotype availability and removing all related individuals, 3655 individuals remained, with a minimum age of 66 and a maximum of 90. The number of cases and controls for each analysis/dynapenia definition is as follows: for FNIH, 351 cases and 3,304 controls; for EWGSOP, 939 cases and 2,716 controls. Logistic regression analyses were conducted using the '--logistic-snp' option on FAST (version 2.4)(2) with imputed continuous dosage data. Covariates for adjustment included age, sex (except in the sex-stratified analyses), study site and PCs 1-4. Only SNPs with a MAF ≥0.02 were included in association analyses.

Berlin Aging Study II (BASE-II)

BASE-II is a multidisciplinary study initiated in 2009 investigating factors related to human aging (3). All subjects are recruited from the Berlin metropolitan area and underwent an extensive phenotypic assessment, including a 2-day internal medicine examination (follow-up will be completed in 2020). The BASE-II research project (Co-PIs are Lars Bertram, Ilja Demuth, Denis Gerstorf, Ulman Lindenberger, Graham Pawelec, Elisabeth Steinhagen-Thiessen, and Gert G. Wagner) is supported by the German Federal Ministry of Education and Research (Bundesministerium für Bildung und Forschung, BMBF) under grant numbers #16SV5536K, #16SV5537, #16SV5538, #16SV5837, #01UW0808, 01GL1716A and 01GL1716B. Another source of funding is the Max Planck Institute for Human Development, Berlin, Germany. Additional contributions (e.g., equipment, logistics, personnel) are made from each of the other participating sites.

Genome-wide SNP genotyping was performed using the "Genome-Wide Human SNP Array 6.0" [Affymetrix, Inc]) on the full BASE-II dataset (3). For full QC and imputation procedures see (REF: PMID 26821332). Briefly, genome-wide imputation of unobserved genotypes was carried out on the QC'ed data using IMPUTE2 v2.2.2 (https://mathgen.stats.ox.ac.uk/impute/impute_v2.html) based on precompiled 'ALL 1000G Phase1 integrated haplotypes' reference panels (December 2013 release) [14]. A total of 27,213,648 SNPs were imputed, but only autosomal SNPs with an IMPUTE info value ≥0.35 and minor allele frequencies ≥1% were retained for subsequent analyses. Overall, genotype and hand grip strength data were available in 1,531 unrelated (i.e. IBD/IBS sharing >12.5% using –genome in PLINK1.9) BASE-II participants aged 60+. To calculate PCs to be used as co-variates in the genome-wide analyses, LD pruning was applied to pre-imputation genotype data using PLINK (--indep-pairwise 1500 150 0.2) resulting in 117835 SNPs. Eigenvalues and eigenvectors were then calculated using PLINK (–pca). Based on the resulting scree plot, we selected the first 10 PCs as covariates. For the actual GWAS we used SNPTEST (https://mathgen.stats.ox.ac.uk/genetics_software/snptest/snptest.html) v2.5.4-beta3 (-frequentist 1 -method score -cov_names PC1 PC2 PC3 PC4 PC5 PC6 PC7 PC8 PC9 PC10 age -cov_names sex (for combined phenotypes only)).

B-Vitamins for the PRevention Of Osteoporotic Fractures (BPROOF) study

The B-PROOF study is a randomized, placebo-controlled, double-blind trial that studied the effect of vitamin B12 and folic acid supplementation on osteoporotic fractures in 2,919 people of 65 years or over, and having homocysteine levels of 12-50 µmol/L. Participants were recruited between 2008 and 2011 and followed during a 2-3 year period (4). Genotyping was done by using the Illumina Omni-express array. Imputations to HRC1.1 were performed using the Michigan Imputation Server with standard settings. The GWAS analyses were performed using rvtest.

Cardiovascular Health Study (CHS)

The Cardiovascular Health Study is a population-based, prospective cohort study of risk factors for development and progression of CHD and stroke in older adults ages 65 years or older (5). A cohort

of 5,201 non-institutionalized men and women were selected and enrolled from randomly generated Medicare eligibility lists in 4 U.S. communities in 1989-90; an additional 687 predominantly African American participants were recruited and enrolled in 1992-93. Clinic examinations were performed at study baseline, at annual visits through 1999, and again in 2005-2006. Participants were contact by telephone annually between exams, and every 6 months after the exams ended. Multiple physical and biological tests have been performed, including assessment of physical function. The current analysis included 3,061 CHS participants of European ancestry for whom genotype and grip strength data were available.

Blood samples were drawn from all participants at their baseline examination and DNA was subsequently extracted from available samples. Genotyping was performed at the General Clinical Research Center's Phenotyping/Genotyping Laboratory at Cedars-Sinai among CHS participants who consented to genetic testing and had DNA available using the Illumina 370CNV BeadChip system. CHS was approved by institutional review committees at each field center and individuals in the present analysis had available DNA and gave informed consent including consent to use of genetic information.

Imputation to the HRC r1.1 2016 panel was performed on the Michigan imputation server. SNPs were excluded for variance on the allele dosage ≤0.01. The GWAS was conducted using a custom program written in R. Logistic regression models adjusted for age, sex (for the combined analyses), field center, and principal components were fit.

European Prospective Investigation of Cancer (EPIC) Norfolk

The European Prospective Investigation into Cancer and Nutrition (EPIC)-Norfolk study (https://dx.doi.org/10.22025/2019.10.105.00004) is a prospective population-based cohort study which recruited 25,639 men and women aged 40-79 years at baseline between 1993 and 1998 from 35 participating general practices in Norfolk, UK. Individuals attended for a baseline health check including the provision of blood samples for concurrent and future analysis. Further health check visits have been conducted since the baseline visit. Participants have contributed information about their diet, lifestyle and health through questionnaires and health checks over two decades. The Norwich Local Research Ethics Committee granted ethical approval for the study. All participants gave written informed consent. Grip strength measurement was taken at the third (2004-2006), the fourth (2012-2016) and the fifth (2016-2018) health checks. To maximum the sample size, we take grip and related measures from a late health check visit if it is unavailable at previous visit. Related samples, ethnic outliers and participants whose age was less than 60 years old were excluded from further analysis.

DNA has been extracted from all EPIC participants and stored blood has been analysed for an extensive range of classical and novel biomarkers. Samples were genotyped using Affymetrix Axiom array at Cambridge Genomic Services, Department of Pathology, University of Cambridge, UK, and samples were excluded if lower call rate (<95%), or heterozygosity outliers, or gender mismatch, or failed channel contrast (DishQC <0.82), or unusually high number of singleton genotypes, or impossible IBD values. SNPs were removed if lower call rate (<95%), or MAF=0, or failed HWE (p<10-6), or clusters failed Affymetrix SNPolisher standard tests and thresholds, or MAF significantly affected by plate, or duplicates or unflippable. A total of 21044 samples were forwarded to

imputation using HRC r1 reference panel, and using UK10K+1000Gp3 reference panel for additional SNPs.

GWAS analysis was performed on 7508 participants using SNPTEST v2.5.4-beta3 (-method newml) under an additive model with covariates age, sex, and the first 10 principal components.

Framingham Heart Study

The Framingham Heart Study (FHS) is a single-site, community-based cohort study that was initiated in 1948 to investigate risk factors for cardiovascular disease and other major illnesses. The FHS includes three generations: the original cohort followed since 1948 (Gen1) (6); their offspring and spouses of the offspring, followed since 1971 (Offspring or Gen2) (7); and children of the offspring enrolled in 2002 (Third Generation or Gen3) (8). The Original cohort enrolled 5,209 men and women who comprised two-thirds of the adult population then residing in Framingham, MA, USA. The Offspring cohort comprises 5,124 persons who have been examined about every 4 to 8 years. Examination 1 of the Gen3 occurred between 2002 and 2005 and involved 4,095 participants. Offspring spouses not previously enrolled who were a biological parent of a Gen 3 participant were enrolled into the New Offspring Spouse cohort to complete family pedigrees. All cohorts continue under active surveillance. The FHS follows two multi-ethnic cohorts, Omni group 1 and Omni group 2 to reflect the current diversity of the town of Framingham, MA.

In the 1990s and early 2000s, DNA samples were collected in the Original, Offspring, Third Generation, and Offspring Spouse cohorts of the FHS. All individuals provided consent for genotyping. NHLBI funded genotyping using 550,000 SNPs (Affymetrix 500K mapping array plus Affymetrix 50K supplemental array). Imputations were performed with miniMACH3 using the HRC release 1.1 reference panel for SNPs passing the following criteria: call rate ≥ 97%, pHWE ≥ 10-6, < 100 Mendel errors, and MAF ≥ 1%.

Grip strength was recorded by trained technicians at the time of Offspring exam 7, exam 8 and exam 9 and Gen 3 exam 2, using a Jamar dynamometer. The maximum of six trials (three trial in each hand) was used. For participants with hand grip data at more than one examination, the first exam the individual was age 60 or older was used for analysis. The Boston University Medical Campus IRB approved the content of all examinations and participant provided informed consent at all attended exam.

Health and Retirement Study (HRS)

The Health Retirement Study is representative American cohort with participants over age 50 to monitor factors related to aging and retirement (9,10). A random subset of ~26,000 participants were selected in three phases (phase 1 in 2006, phase 2 in 2008, and phase 3 in 2010) to collect biological specimen between 2006 and 2010. The DNA samples were genotyped at the Center for Inherited Disease Research (CIDR) on Illumina HumanOmni2.5 array, Phases 1-2 on HumanOmni2.5-4v1 and Phase 3 on HumanOmni2.5-8v1. Both arrays are designed to human genome build 37. Imputation analyses were performed using IMPUTE version 2.3 (11).

Genotyping data were available for 15,708 samples (6519 males and 9189 females) in the combined Phase 1-3 dataset. 15,454 remained after filtering related or duplicated samples, genotyping controls, and those with a missing call rate ≥ 2%. 12,940 (84%) were Whites. Of which, 10,919 reached 60 years old. The grip strength was measured using a medley spring-type hand dynamometer. Two measurements were taken from each hand and the maximum at the most recent visit was used to conduct association analyses. Grip strength ≥ 90 kg was considered unreal and set to missing based on actual data distribution. Dynapenia was determined based on the EWGSOP or FNIH definition. 10,814 white participant (4650 males and 6164 females) had both genotyping and grip strength data. 3624 (34%) and 1754 (16%) met the EWGSOP and FNIH criteria, respectively. Women were more likely to have dynapenia than men by the EWGSOP (39% vs. 27%) or FNIH (18% vs. 14%).

In genome-wide association analyses, the EWGSOP or FNIH dynapenia was associated with SNPs, one at a time, in a linear mixed effects model, including additive allelic effect of the candidate SNP, sex (if not sex-specific), age at measurement, plus random polygenic and environment effects. The analyses were conducted using the BOLT-LMM version 2.2 (12), with precomputed LD scores from Europeans in the 1000 Genomes.

InCHIANTI study

The InCHIANTI study is a population-based epidemiological study aimed at evaluating the factors that influence mobility in the older population living in the Chianti region in Tuscany, Italy. The details of the study have been previously reported (13). Briefly, 1616 residents were selected from the population registry of Greve in Chianti (a rural area: 11,709 residents with 19.3% of the population greater than 65 years of age), and Bagno a Ripoli (Antella village near Florence; 4,704 inhabitants, with 20.3% greater than 65 years of age). The participation rate was 90% (n=1453), and the subjects ranged between 21-102 years of age. Overnight fasted blood samples were for genomic DNA extraction. Maximal isometric hand grip strength was measured in kilograms using a hand-held dynamometer (Smith & Nephew, Agrate Brianza, Milan, Italy). The subject was seated in front of a bench with the tested arm supported on the bench and the elbow flexed to 45°. Participants were asked to perform the task twice with each hand, and the maximum strength attained during the four trials was used for the present analyses. Dynapenia was determined using the FNIH criteria and EWGSOP criteria. The study protocol was approved by the Italian National Institute of Research and Care of Aging Institutional Review and Internal Review Board of the National Institute for Environmental Health Sciences (NIEHS). All participants provided written informed consent.

Genome-wide genotyping was conducted using Illumina Infinium HumanHap 550K SNP arrays (14). Genotyping was completed for 1210 subjects with a sample call rate >97%, heterozygosity rates > 0.3 and correct sex specification. 495,343 autosomal SNPs that passed quality control (MAF>1%, completeness >99%, HWE > 10-4) were used for imputation using as reference the Haplotype Reference Consortium (HRC) panel, version 1.1. The imputation was done using Minimac, and the process was facilitated by the Michigan Imputation server (15). Associations between grip strength and SNP dosages was performed using logistic regression in PLINK 1.9, adjusting for age, sex (except sex-specific analyses), study site, and 10 principal components.

LASA: Longitudinal Aging Study Amsterdam

Longitudinal Aging Study Amsterdam (LASA) is an ongoing, population-based cohort of individuals 55 years and older living in the Netherlands. The design and rationale is described elsewhere (16,17). In short, 3017 participants (55-84 years old) were included at baseline (1992-1993) and two additional cohorts were added in 2002-2003 and 2012-2013 with respectively 1002 and 1023 participants. Follow-up visits were conducted every 3 years. Trained interviewers collected data on cognitive, emotional, physical and social functioning during a home interview. Subsequently, all participants were invited for a medical interview during which further diagnostic examinations were done and blood samples were drawn. LASA has been approved by the Medical Ethics Committee of VU University Medical Center. All participants gave written informed consent.

DNA was isolated from buffy coats or full blood samples drawn at baseline. Genotyping was done using two arrays: Axiom-NL Array (Affymetrix Inc, Santa Clara, CA., USA) and Infinium Global Screening Array (GSA) (Illumina Inc, San Diego, CA., USA). Quality control (QC) was done separately for each array using Ricopili (Rapid Imputation for COnsortias PIpeLIne for GWAS), an established tool developed by the Psychiatric Genomics Consortium (18). After QC, the data was imputed using as reference the Haplotype Reference Consortium (HRC) panel, version 1.1 (19). The imputation was done using Minimac 3, and the process was facilitated by the Michigan Imputation server(20).

Grip strength was measured during the medical interview using a dynamometer (Takei TKK 5001, Takei Scientific Instruments Co. Ltd., Tokyo, Japan) and it was recorded in the nearest 1 kg. Dynapenia was determined using the FNIH criteria and EWGSOP criteria.

The analyses were performed separately per genotyping array and were adjusted for age, sex (except sex-specific analyses), 10 principal components and cohort (for the GSA array). Logistic regression in Plink v.1.9 was used.

Long-Life Family Study

The Long Life Family Study is a multi- centre international study on the genetics and familial components of exceptional survival. Between 2006 to 2009, LLFS successfully enrolled and rigorously phenotyped probands, their siblings, and children of families demonstrating exceptional survival (4,953 individuals from 539 families; N=1727 probands; 3226 offspring). LLFS includes: Field centres at Boston University, Columbia University, University of Pittsburgh, and University of Southern Denmark, at Laboratory Core (University of Minnesota) and a Coordinating Center (Washington University in St. Louis). In brief, the LLFS recruited selected families with multiple exceptionally old living individuals, the probands were ≥79 years old in the USA, and ≥90 years old in Denmark. Families were selected to participate in the study based on The Family Longevity Selection Score (FLoSS) (21) which calculated the rank sibships by current age or age at death of siblings, the size of the sibship and the number of alive individuals available for study. A proband's family was eligible if the FLoSS reached a score of 7 or higher, which met the following criteria: (1) the proband, at least one living sibling, and one of their living offspring (minimum family size of 3) were all able to give informed consent, and (2) were willing to participate in the interview and examination including the blood sample for serum and DNA extraction. The age of the 3359 participants who were aged 60 years and older with valid grip strength and genotyped data in the current analyses was: 77.6 ± 12.9 (range: 60-110).

Grip strength was measured using a JAMAR® hand-held dynamometer (Sammos Preston Rolyan, Bolingbrook, IL). Two trials for each were conducted, and the maximum value for the strongest hand was used in analyses.

Genotype data were produced on 4,716 of the consented LLFS subjects using the Illumina 2.5 million HumanOmni array, with the purpose of utilizing them in genome-wide association (GWA) analyses. Genotypes were called using Bead Studio. LLFS QC of the genotypes included using the package GRR (Graphical Representation of Relationships)(22) to check familial relationships and sample switches based on Identity-by-State genotypes. Corrections to familial relationships were made as warranted by the data. After GRR, additional QC included removing 18 samples with autosomal call rates less than 97.5%, leaving 4,693 (4,597 with EU ancestry) subjects for further analysis. To determine and exclude outliers with respect to ancestral population, we generated principle components (PCs) with smartpca2016 (23) from 112,639 Tag SNPs in 4,597 (of 4,693) LLFS samples of European Ancestry and included 2,504 haplotypes from 1000 genomes Cosmopolitan panel to support ancestral clustering. 18 additional individuals of the 4,597 samples with EU ancestry were identified as PCs outliers and removed prior to imputation. Further, we calculated sample heterozygosity and removed 5 outliers (3 overlap in PCA outliers) with excess heterozygosity, i.e. heterozygosity > median + 3*IQR, thereby leaving 4,577 samples for imputation.

SNP QC included using Loki (24) to identify all Mendel errors in SNPs among families. 3,647 SNPs were identified as outliers with respect to total number of detected Mendelian errors and were excluded. All other genotype calls resulting in Mendel errors were set to missing, which occurred 153,363 times in our data. SNPs with a call rate lower than 98% were also excluded (n=83,774; 1,188 of these were also Mendelian outlier SNPs). Applying both the call rate and Mendelian error criteria, 86,233 autosomal SNPs were removed, leaving 2,225,478 SNPs in the cleaned genotyped data passing QC criteria.

From the SNPs that passed LLFS QC procedures, we applied the following criteria, per the MI server, to ensure the highest quality imputation. The imputation 'scaffold' for the MI server included SNPs from our cleaned genotyped SNPs (n=2,225,478) which met the following additional criteria: 1) Hardy-Weinberg equilibrium (p>=1E-06); 2) No allele mismatch when compared with 1000HG; 3) No position mismatch when compared with 1000HG (e.g. assembly GRCh37); 4) No association with known inversions between GRCh37 and GRCh38 (identified by order reversal between rs-named SNPs in b138 and b144 from NCBI annotation downloads); and 5) Monomorphic markers.

The cleaned binary files (one for each chromosome) were then uploaded to the imputation server for pre-phasing and imputation in VCF format (after bgzip and tabix in the UNIX environment). The final number of clean autosomal SNPs used for imputation was 1,559,272. Autosomal Imputation was performed on the Michigan Imputation Server (https://imputationserver.sph.umich.edu) by chromosome (e.g. 22 simultaneous jobs). The reference panel used was HRC r1.1 2016, phasing was performed with Eagle v2.3, population was set to EUR., and the mode option employed quality control and imputation.

GWAS was done using R package GENESIS via Bioconductor. The protocol in GENESIS is to first fit a null model without genotype data, which adjusts the outcome based on the covariates, principal components, and family correlations (GENESIS function 'fitNullModel'). Genetic relatedness matrix is established using sample of independent genotyped SNPs, then specified as the covariance matrix in the mixed model. Population stratification is adjusted for by a set of principal components specific to the outcome, which are derived from the same sample of independent SNPs. This data is then used in SNP association tests (GENESIS function 'assocTestSingle'), so that the mixed models only need to

be run once. Tests were adjusted for Age, sex (if combined sex analysis), field centre, principal components (for population stratification). Betas, SEs, and Odds ratios were separately calculated from the Score and Score SE statistics output by GENESIS using the following equations: (https://support.bioconductor.org/p/119621/)

(1) $Beta = \frac{Score}{Score.SE^2}$

(2) $SE = \frac{1}{Score.SE}$

(3) $OR = \exp(Beta)$

MrOS (Osteoporotic Fractures in Men) study

The Osteoporotic Fractures in Men (MrOS) study is a multicenter, prospective study including older men in Sweden, Hong Kong and the United States. The MrOS Sweden study (n=3014) consists of three sub-cohorts from three different Swedish cities (n=1005 in Malmö, n=1010 in Gothenburg, and n=999 in Uppsala) (25). Study subjects (men aged 69 to 81 years) were randomly identified using national population registers. A total of 45% of the subjects who were contacted participated in the study. To be eligible for the study, the subjects had to be able to walk without assistance, provide self-reported data, and sign an informed consent. The study was approved by the ethics committees at the Universities of Gothenburg, Lund, and Uppsala. Informed consent was obtained from all study participants. In this study 941 unrelated participants from Gothenburg and 891 unrelated participants from Malmö were included.

A Jamar® hydraulic hand dynamometer (5030J1, Jackson, MI, USA), with adjustable handgrip, was used in the grip strength test. Participants were made to sit in a standard chair with the arm resting on a moveable table with the dynamometer in an upright position. Two trials were performed on each hand. The better of the two results (presented as kilograms of force) was used in the analyses. Grip strength was not measured if the subject had current arthritis or pain in the wrist or hand or had undergone fusion, arthroplasty, tendon repair, synovectomy or related surgery of the upper extremity in the 3 months preceding the test. The coefficient of variation was 0.5%.

Genotyping, imputation and quality controls of MrOS Gothenburg were performed using the Illumina HumanOmni1_Quad_v1-0 B array. Genotypes were called using the Illumina's BeadStudio calling algorithm. The sample quality control exclusion criteria were sample call rate < 97%, excessive autosomal heterozygozity, first and second degree relatives, genotypic sex mismatch using X and Y chromosome probe intensities and gross chromosome abnormalities. Genotyped SNPs with GenTrain scores <0.6, cluster separation scores <0.4, call rates <97%, or MAF <0.01 were excluded. Also, autosomal SNPs with Hardy-Weinberg Equilibrium P-value <10-4 were excluded and genotype clusters for SNPs on chrX, chrY, chrXY and chrMT were reviewed manually. 714543 autosomal SNPs passed quality control.

Genotyping, imputation and quality controls of MrOS Malmö were performed using the HumanOmniExpress-12v1_B build 36. The sample quality control exclusion criteria were sample call rate < 97.5%, missing data, excessive autosomal heterozygozity, familiar relationship (one sample

excluded), genotypic sex mismatch, non-caucasians and gross chromosome abnormalities. SNPs with call rates<95% were excluded. 725409 autosomal SNPs passed quality control.

The genotype data for both MrOS Gothenburg and MrOS Malmö were pre-phased first without a reference panel, using SHAPEIT2. The imputation was done using Sanger Imputation Service to the Haplotype Reference Consortium release 1.1.

Associations between grip strength and SNP dosages were obtained using logistic regression in PLINK 1.9, adjusting for age.

ROSMAP: the Religious Orders Study (ROS) and Memory and Aging Project (MAP)

The ROS, started in 1994, enrolled Catholic priests, nuns, and brothers, from about 40 groups in 12 states (26). The follow-up rate of survivors exceeds 90%. Participants were free of known dementia at enrollment, agreed to annual clinical evaluations, and signed both an informed consent and an Anatomic Gift Act form donating their brains at time of death. Participants take a neuropsychological test battery. DNA was extracted from whole blood, lymphocytes, or frozen post-mortem brain tissue. Genotyping was performed at the Broad Institute's Center for Genotyping and the Translational Genomics Research Institute and the Children's Hospital of Philadelphia.

The Rush Memory and Aging Project, started in 1997, enrolled older men and women from assisted living facilities in the Chicago area with no evidence on dementia at baseline (26). The follow-up rate of survivors exceeds 90%. Participants agreed to annual clinical evaluations, and signed both an informed consent and an Anatomic Gift Act form donating their brains at time of death. Participants were invited to take a neuropsychological test battery. DNA was extracted from whole blood, lymphocytes, or frozen postmortem brain tissue. Genotyping was performed at the Broad Institute's Center for Genotyping and the Translational Genomics Research Institute and the Children's Hospital of Philadelphia.

Rotterdam Study

The Rotterdam Study is an ongoing prospective population-based cohort that investigates occurrence, determinants, and consequences of diseases in an ageing population (27). The first baseline measurement of Rotterdam Study started in 1990. After two expansions in 2000 and 2006, it comprised 14,926 participants aged 45 years and over by the end of 2008. Follow-up visits were held every 3-5 years. The Rotterdam Study has been approved by the Medical Ethics Committee of the Erasmus MC (registration number MEC 02.1015) and by the Dutch Ministry of Health, Welfare and Sport. All participants provided written informed consent to participate in the study and to have their information obtained from treating physicians. Imputations to HRC1.1 were performed using the Michigan Imputation Server with standard settings (15,19).

Study of Health in Pomerania (SHIP)

The Study of Health in Pomerania (SHIP) is a population-based project in West Pomerania, the north-east area of Germany (28). A sample from the population aged 20 to 79 years was drawn from

population registries. First, the three cities of the region (with 17,076 to 65,977 inhabitants) and the 12 towns (with 1,516 to 3,044 inhabitants) were selected, and then 17 out of 97 smaller towns (with less than 1,500 inhabitants), were drawn at random. Second, from each of the selected communities, subjects were drawn at random, proportional to the population size of each community and stratified by age and gender. Only individuals with German citizenship and main residency in the study area were included. Finally, 7,008 subjects were sampled, with 292 persons of each gender in each of the twelve five-year age strata. In order to minimize drop-outs by migration or death, subjects were selected in two waves. The net sample (without migrated or deceased persons) comprised 6,267 eligible subjects. Selected persons received a maximum of three written invitations. In case of non-response, letters were followed by a phone call or by home visits if contact by phone was not possible. The SHIP population finally comprised 4,308 participants (corresponding to a final response of 68.8%). The medical ethics committee of the University of Greifswald approved the study protocol, and oral and written informed consents were obtained from each of the study participants. Handgrip strength was assessed in the second five-year follow-up of the study (SHIP-2) and used for this project.

Nonfasting blood samples were drawn from the cubital vein in the supine position. The samples were taken between 07:00 AM and 04:00 PM, and serum aliquots were prepared for immediate analysis and for storage at -80 °C in the Integrated Research Biobank (Liconic, Liechtenstein). The SHIP samples were genotyped using the Affymetrix Genome-Wide Human SNP Array 6.0. Hybridisation of genomic DNA was done in accordance with the manufacturer's standard recommendations. Genetic data were stored using the database Caché (InterSystems). Genotypes were determined using the Birdseed2 clustering algorithm. For quality control purposes, several control samples where added. On the chip level, only subjects with a genotyping rate on QC probesets (QC callrate) of at least 86% were included. Finally, all arrays had a sample callrate > 92%. The overall genotyping efficiency of the GWA was 98.55 %. Imputation of genotypes was performed using the HRCv1.1 reference panel and the Eagle and minimac3 software implemented in the Michigan Imputation Server for pre-phasing and imputation, respectively. SNPs with a Hardy-Weinberg-Equilibrium p-value <0.0001, a call rate <0.95, and monomorphic SNPs were removed before imputation. The GWAS were performed sex-stratified using EPACTS-3.2.6-patched adjusting for age, the (two) handgrip measurement devices, and the first 10 genetic principal components.

Toledo Study for Healthy Aging (TSHA)

Data were taken from the Toledo Study for Healthy Aging, a population-based study conducted on 2,488 individuals aged 65 years and older. Study participants were selected by a two-stage random sampling from the municipal census of Toledo, covering both institutionalized and community dwelling persons from rural and urban settings. Data were collected from 2006 to 2009, and included information on social support, activities of daily living, comorbidity, physical activity, quality of life, depressive symptoms, and cognitive function. In addition, a nurse collected anthropometric data, conducted tests of physical performance (walk speed, upper and lower extremities strength, and the stand-and-sit from a chair test) and obtained a blood sample. The diagnosis of the frailty syndrome was based on the Fried criteria (weakness, low speed, low physical activity, exhaustion, and weight loss). For this analysis, participants from the second wave of data collection 2013 – 2015 were included.

Genotyping was performing using Illumina Infinium Global Screening Array. The call rate was 0.98, 3.4% of the sample presented a call rate lower than 95% and were excluded from the analysis.

Imputation Panel: HRC (Version r1.1 2016) using Michigan Imputation Server. GWAS was performed using SNPTEST logistic regression adjusted for age, sex, and 6 EVs.

UK Biobank (UKB)

Between 2006 and 2010, 503,325 volunteers (aged 40 to 70 years old) were recruited from across the United Kingdom to the UK Biobank study. Genetic data was available on 488,377 UK Biobank participants after genotype calling and quality control performed centrally by the UK Biobank team (29). We selected 451,447 participants identified as 'white European' through self-report and verified through principal components analysis based on genotypes. Briefly, principal components were generated in the 1000 Genomes Cohort using high-confidence SNPs to obtain their individual loadings. These loadings were then used to project all of the UK Biobank samples into the same principal component space and individuals were then clustered using principal components 1 to 4 (see (30) for details). Imputation of 39,235,157 genetic variants from the Haplotype Reference Consortium panel was performed using IMPUTE4 centrally by the UK Biobank team (29). After filtering for variants with MAF ≥0.1%, missingness <1.5%, imputation quality >0.1 and with Hardy-Weinberg equilibrium (HWE) $P>1x10^{-6}$ within the European-descent participants 11,516,125 imputed autosomal variants were eligible for the analyses. We used BOLT-LMM v2.3.2 to model the associations between imputed variants (dosages) and each phenotype (12) using LD Score provided with the package for European populations. Although this means we have not used a logistic regression model, this approach has the advantage that the linear mixed effects model approach robustly accounts for relatedness and population structure.

Wisconsin Longitudinal Study (WLS)

The Wisconsin Longitudinal Study (WLS) is a one-third sample of all 1957 Wisconsin high school graduates and a randomly selected sibling (31). These respondents were originally empaneled with an in-person questionnaire at age 18 (1957), which was followed with a mail survey of parents in 1964, telephone survey in 1975, mail and telephone surveys in 1993 and 2004 and in-person interviews in 2011, where data on grip strength was collected from participants. The WLS has a high response rate, exceeding 80 percent in most rounds of data collection. Between In 2006-11, the WLS collected saliva samples from respondents using Oragene kits (32). After quality control, a total of 9,012 graduate and sibling respondents were genotyped at ~710,000 markers (before imputation) utilizing the Omni-Express beadchip. Genotyping was complete at Johns Hopkins' Center for Inherited Disease Research (CIDR) and data cleaning was performed in collaboration with the Genetic Analysis Center at the University of Washington. The detailed procedures employed to generate the genetic data are available on the WLS website (https://www.ssc.wisc.edu/wlsresearch/documentation/GWAS). Genotype imputation using the Haplotype Reference Consortium (HRC) v1.1 panel was performed.

In the sample aged 60 or over at grip assessment (n=7,190) BOLT-LMM was used to model associations between the imputation variants and low grip strength phenotypes. Covariates used were age at grip strength measurement, age at DNA collection, 10 principal components, and sex.

WLS Data, documentation and other material are accessible at http://www.ssc.wisc.edu/wlsresearch/.

GWAS meta-analysis methods

Each cohort used the optimum GWAS method available at the time. Most used logistic regression models or equivalent to derive Odds Ratios. Some (HRS, UK Biobank and WLS) were able to use BOLT-LMM to maximise discovery power by including all related participants (appropriate adjustment made in the Linear Mixed Model methods but larger sample sizes are required for the method to be robust). While BOLT-LMM was developed for quantitative traits, it can be used for binary traits as long as they are sufficiently balanced (cases >10% of sample) (33). We therefore converted the regression coefficients from BOLT-LMM to Odds Ratios using a method published by Lloyd-Jones in 2018 (34) prior to meta-analysis. This method uses the genetic effect (the beta), allele frequency, and sample prevalence to estimate the Odds Ratio from the Beta. We used the published R function to perform the transformation from the HRS, UK Biobank and WLS results (https://github.com/lukelloydjones/ORShiny/blob/master/shiny_lmor_func.R). Finally, inverse variance-weighted meta-analysis with genomic control was performed by METAL (35) on the log-Odds Ratios.

Supplementary Figure 1: QQ plot of EWGSOP low grip GWAS results

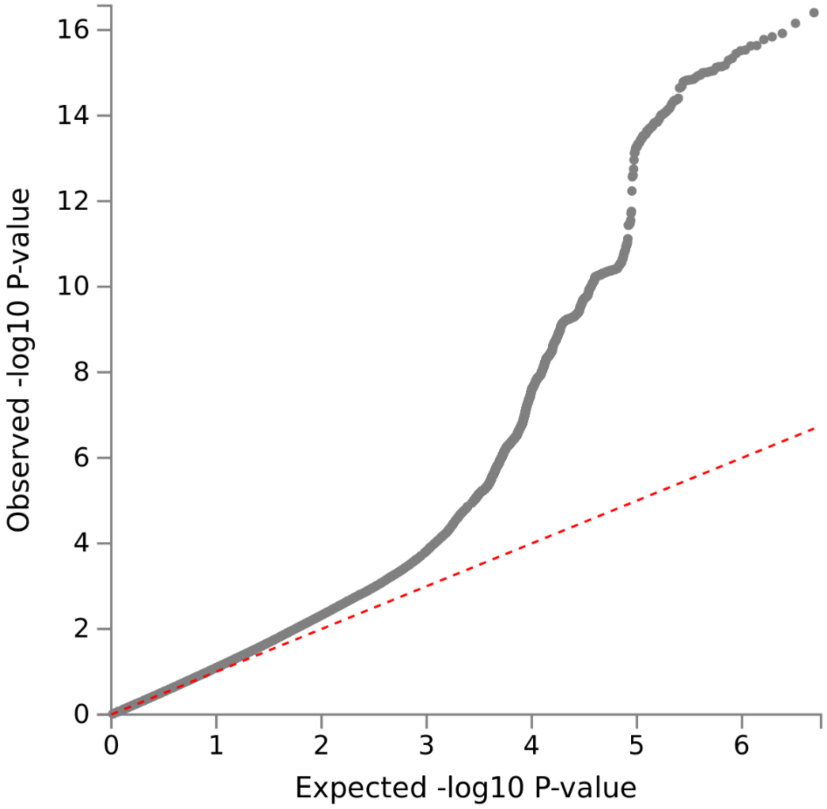

Supplementary Figures 2A-O: LocusZoom plots for the 15 EWGSOP low grip loci

The LocusZoom online tool (http://locuszoom.org) was used to plot the regions around the 15 loci (panels A to O) significantly ($p<5*10^{-8}$) associated with low grip strength in this GWAS meta-analysis. The results can be explored using the below link.
https://my.locuszoom.org/gwas/532795/?token=ad786aade6e44e90935921594de5185b

Supplementary Figure 2A: rs34415150 (chr6:32560477)

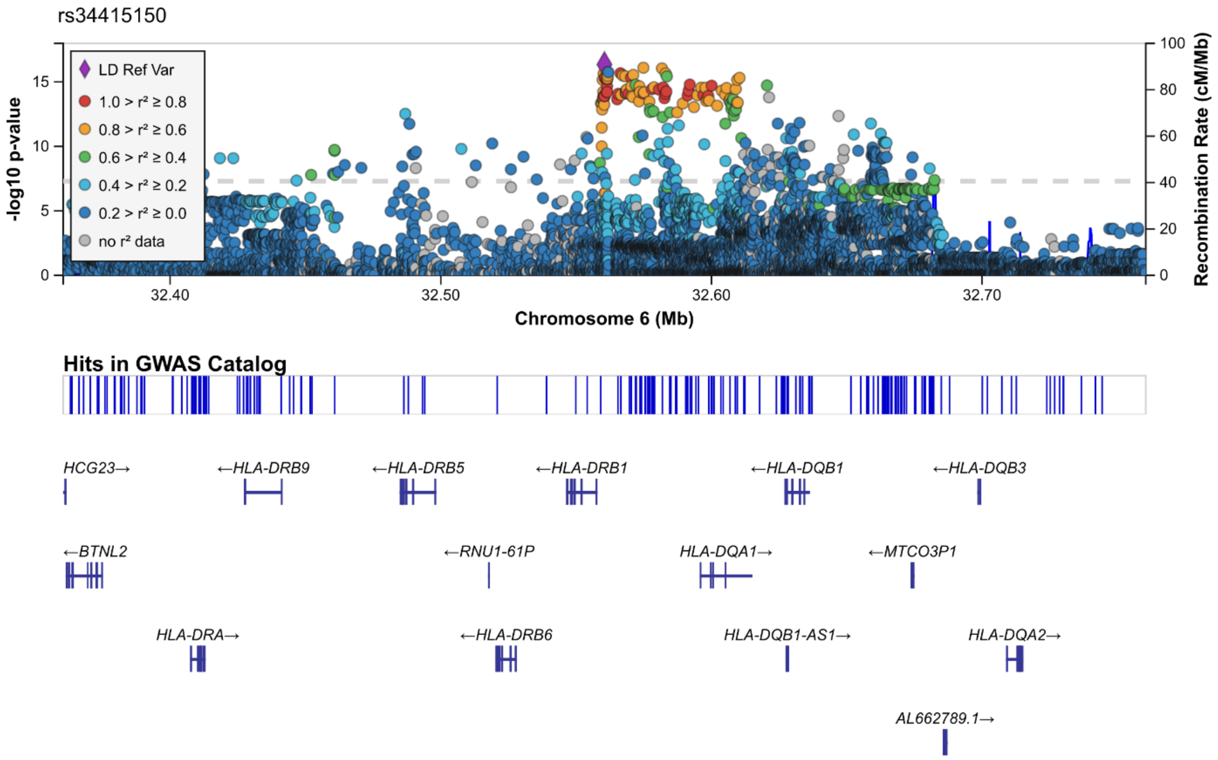

Supplementary Figure 2B: rs143384 (chr20:34025756)

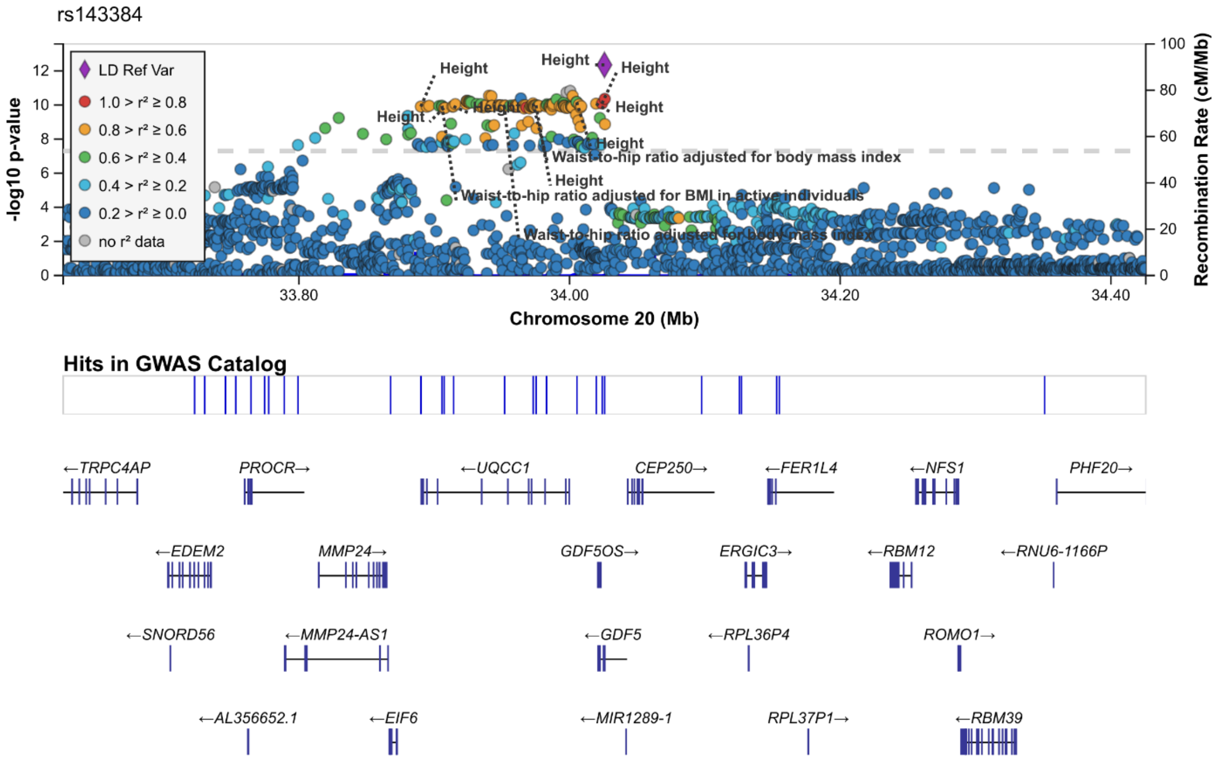

Supplementary Figure 2C: rs62102286 (chr18:46592408)

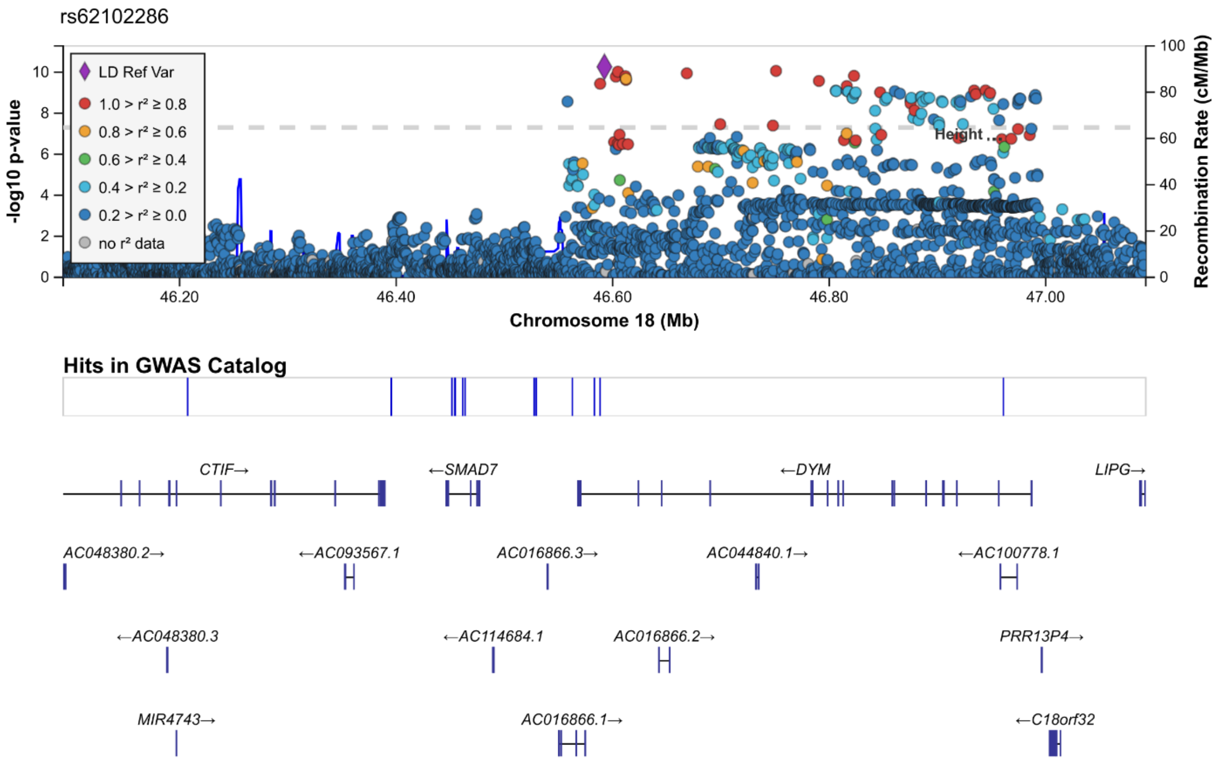

Supplementary Figure 2D: rs3118903 (chr13:51099577)

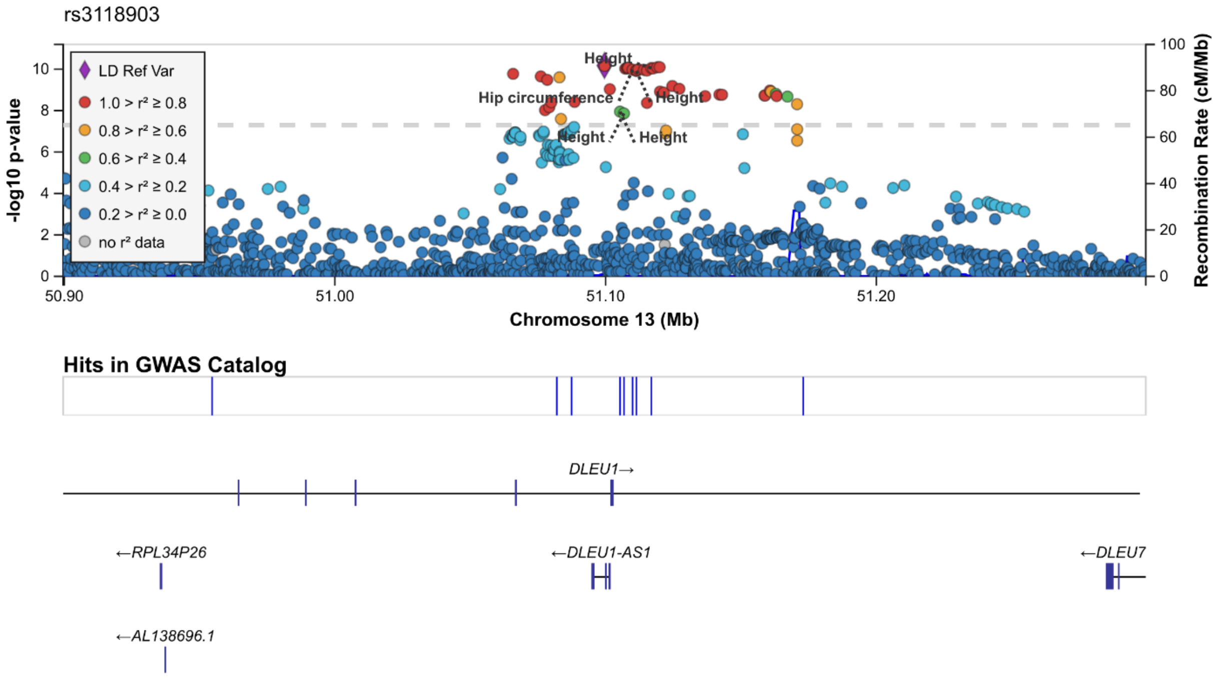

Supplementary Figure 2E: rs13107325 (chr4:103188709)

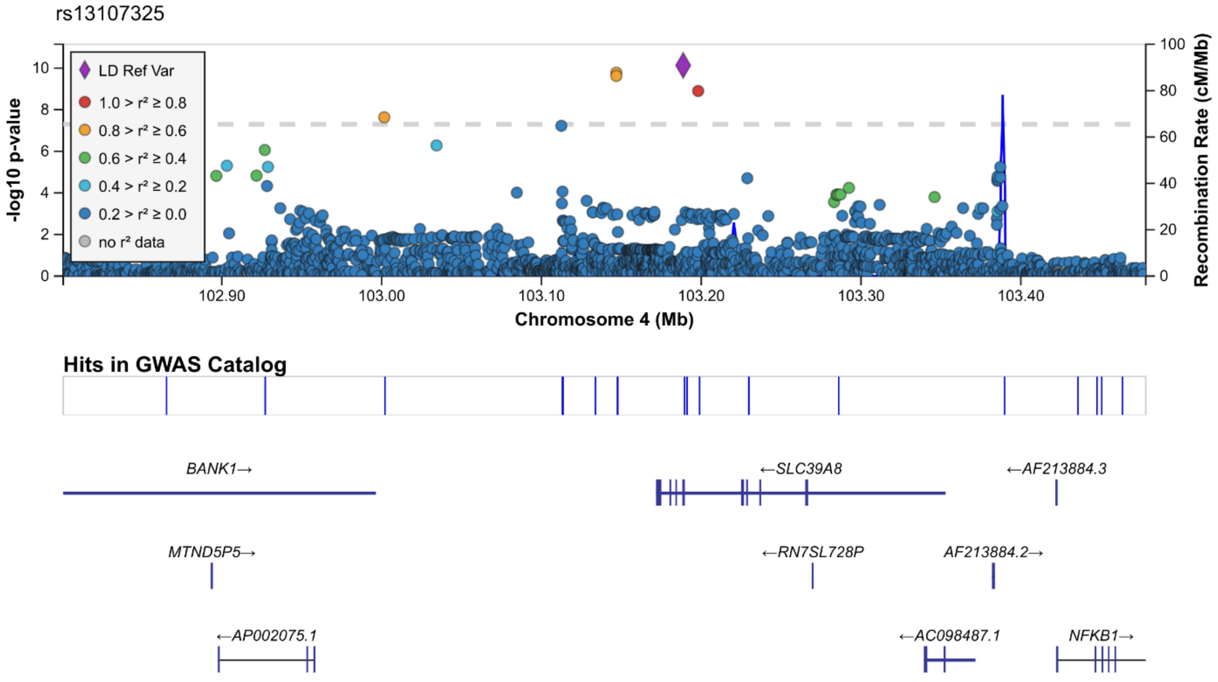

Supplementary Figure 2F: rs11236213 (chr11:74394369)

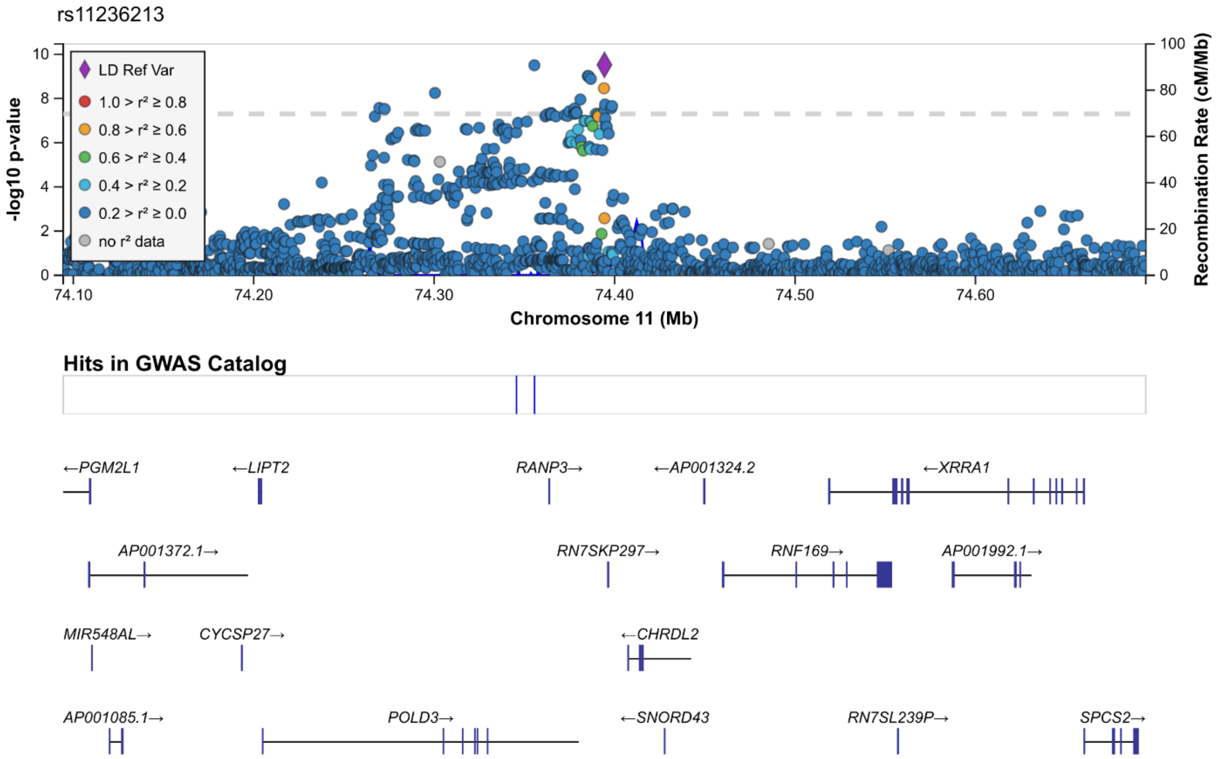

Supplementary Figure 2G: rs34464763 (chr12:15032860)

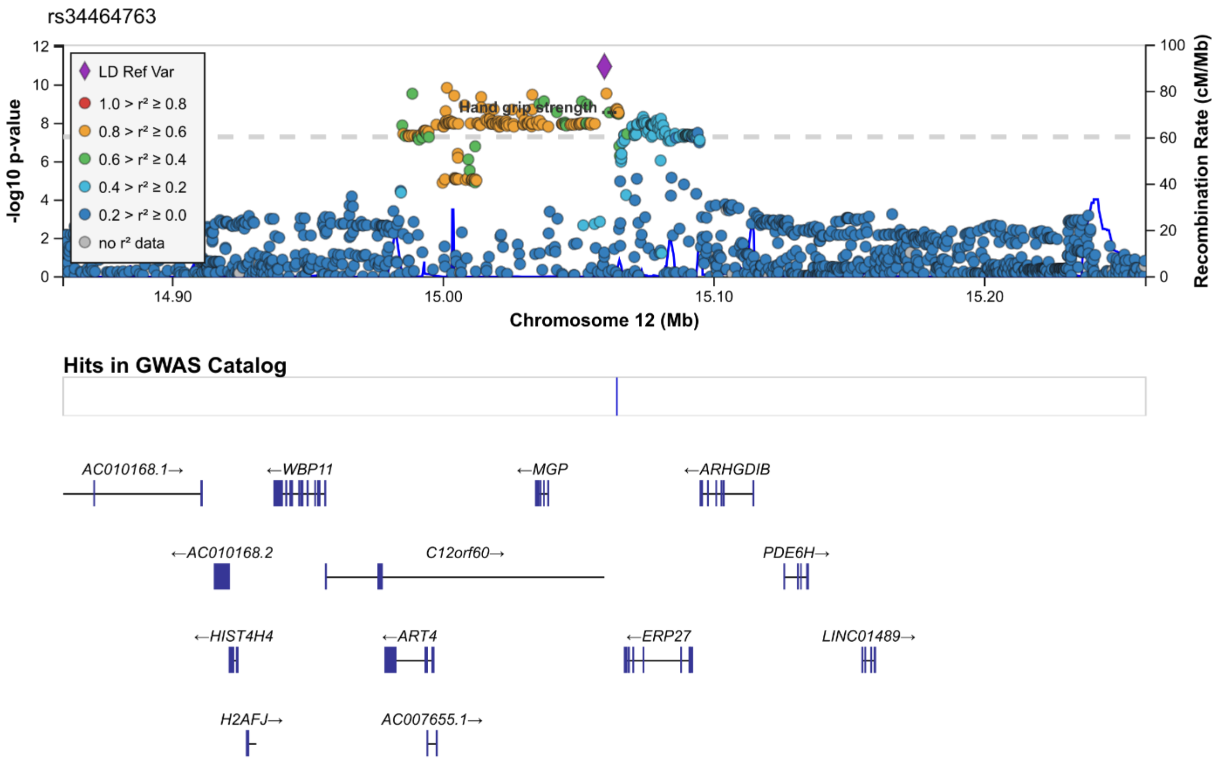

Supplementary Figure 2H: rs143459567 (chr16:24600412)

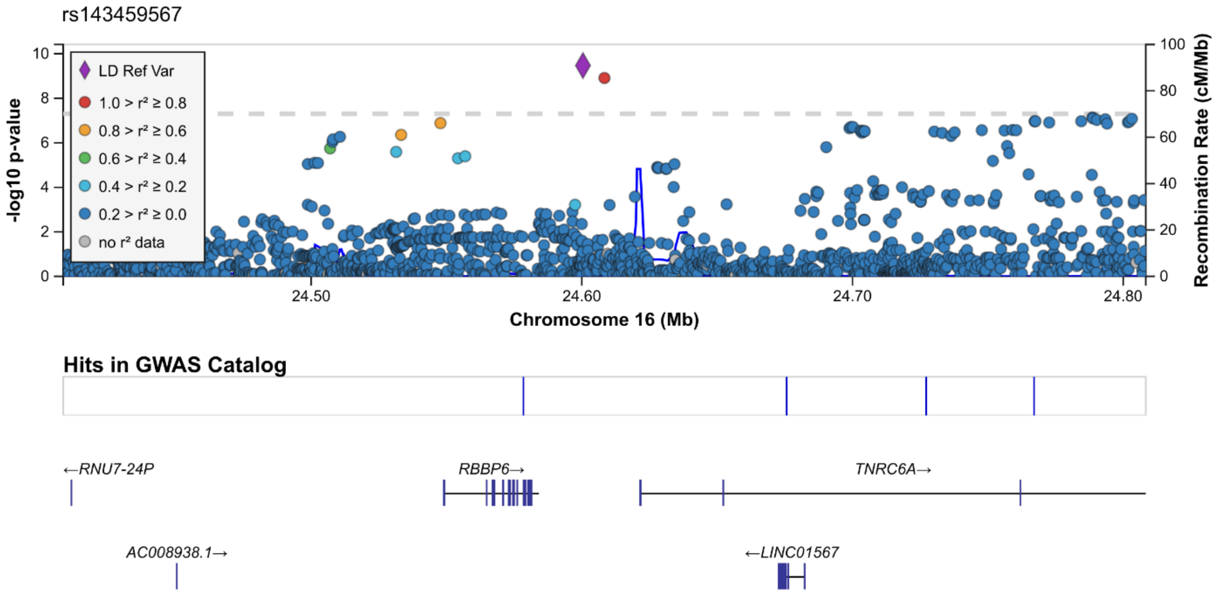

Supplementary Figure 2I: rs2899611 (chr15:58327347)

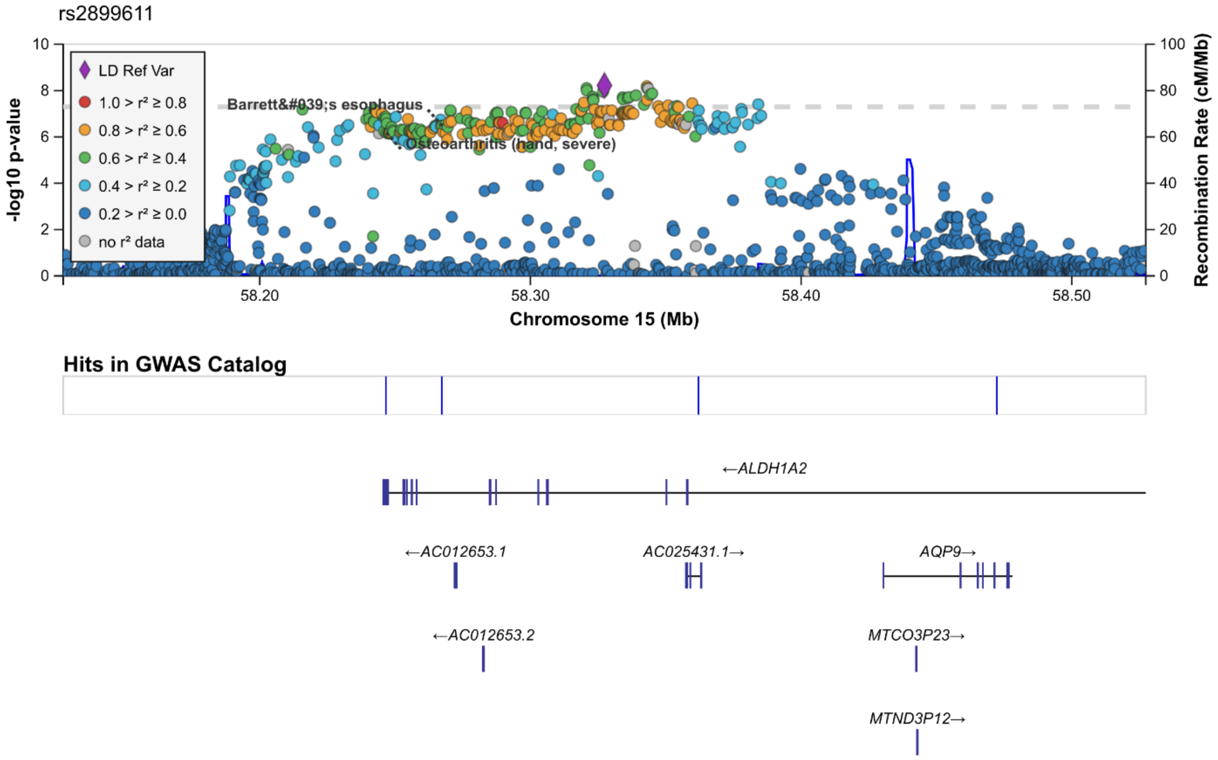

Supplementary Figure 2J: rs958685 (chr2:70703847)

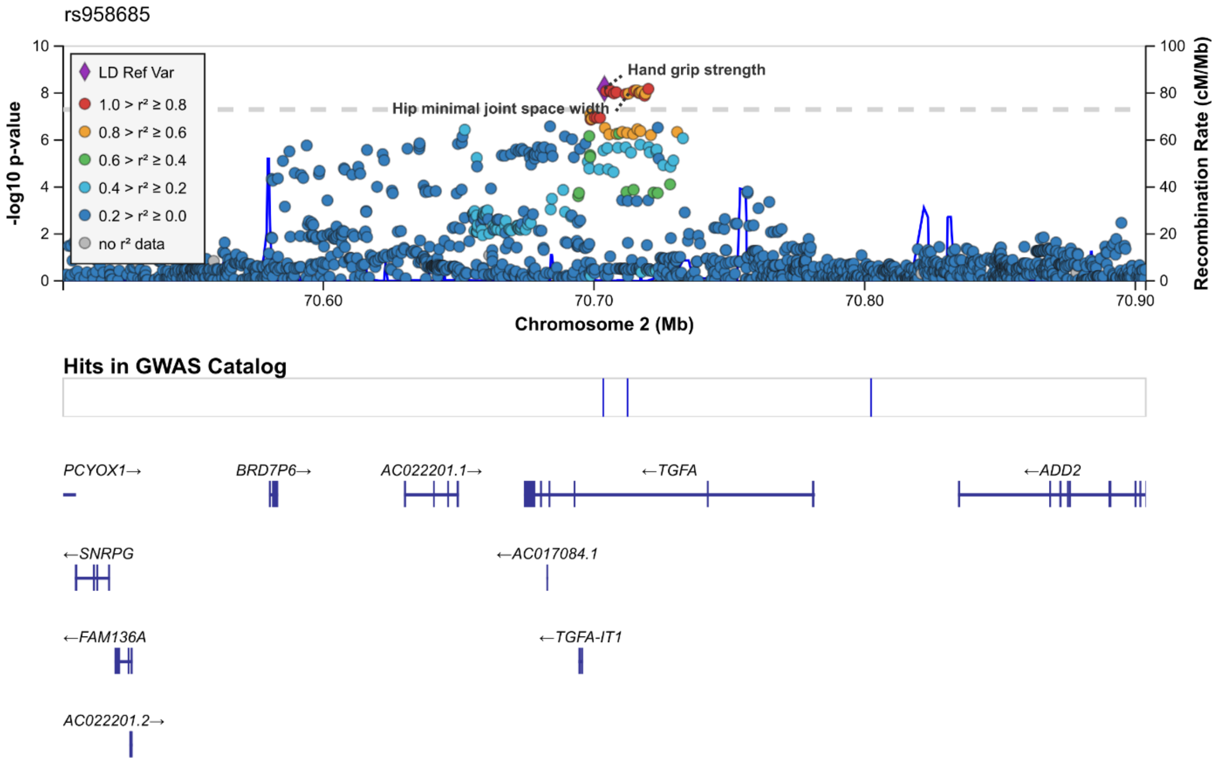

Supplementary Figure 2K: rs7624084 (chr3:141093285)

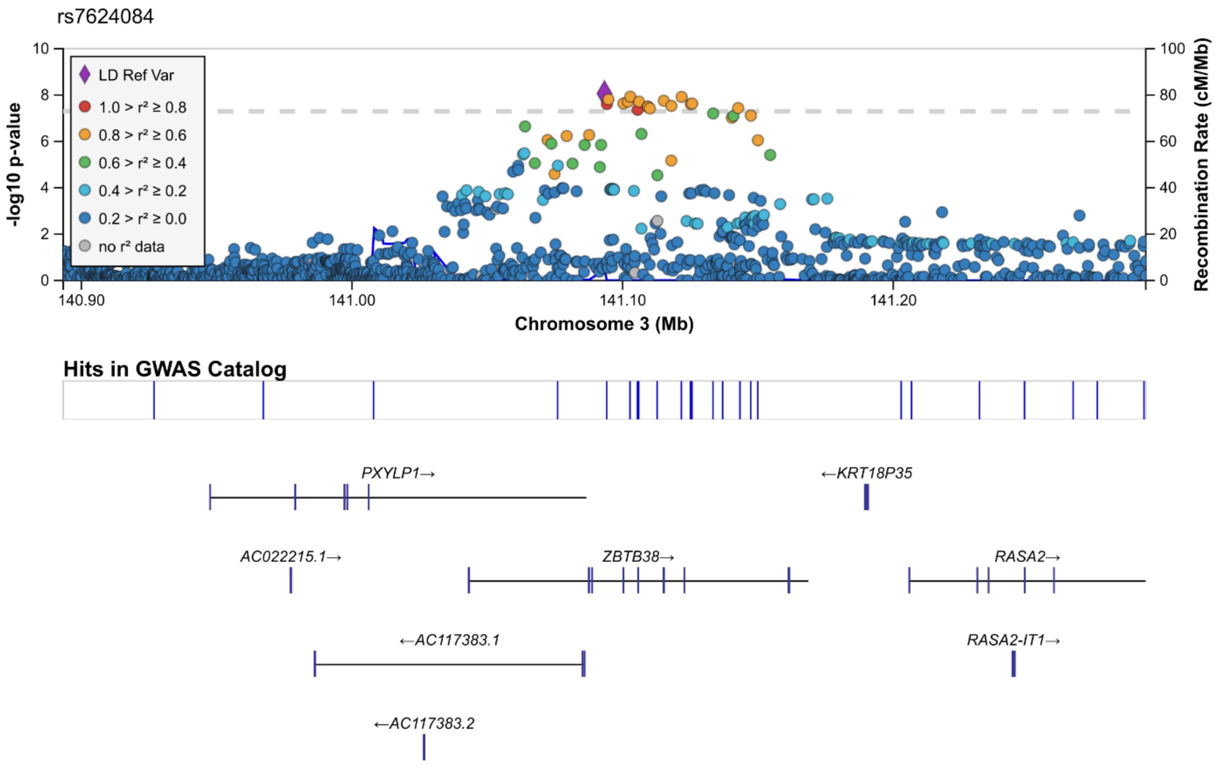

Supplementary Figure 2L: rs79723785 (chr19:55818225)

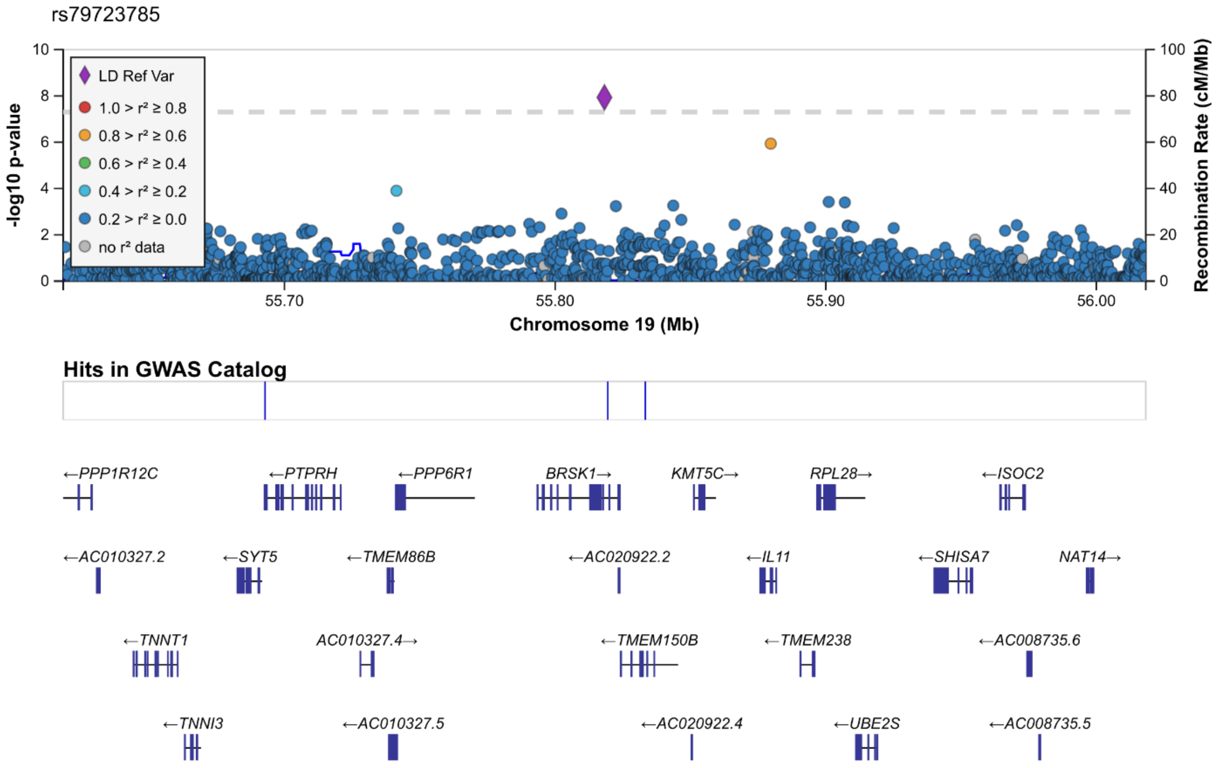

Supplementary Figure 2M: rs10952289 (chr7:150524681)

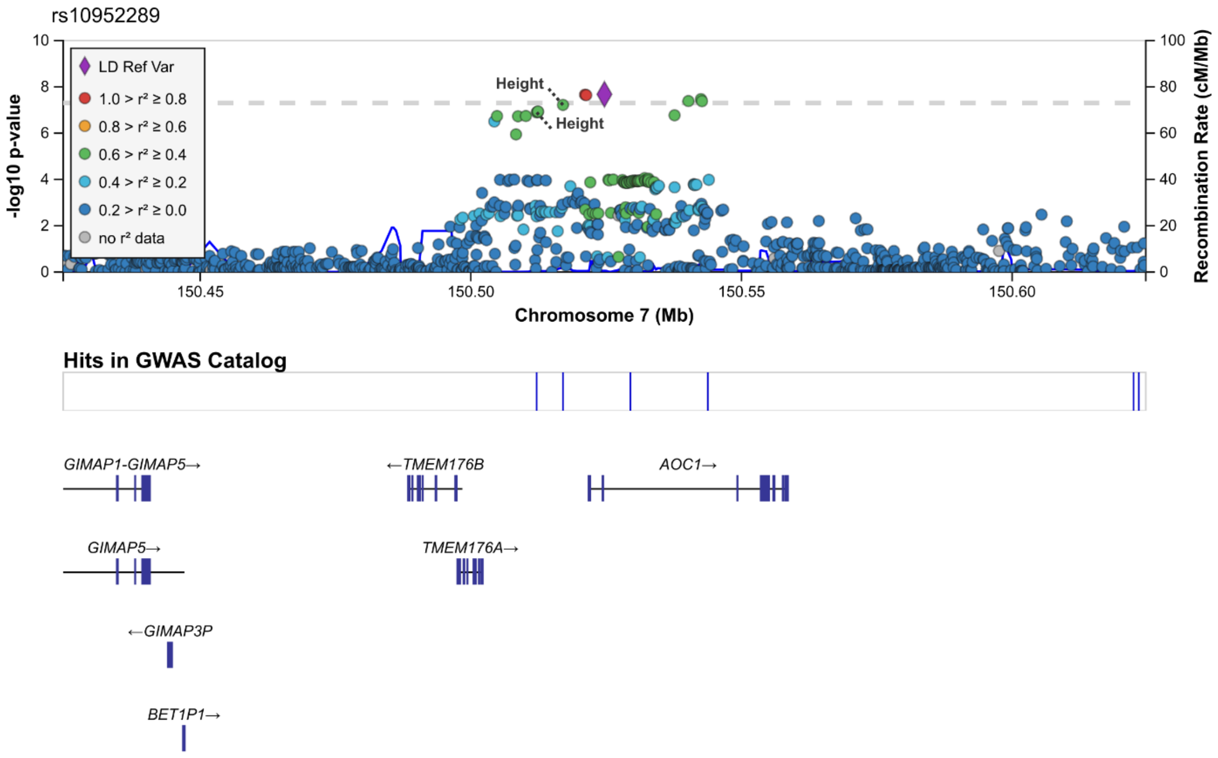

Supplementary Figure 2N: rs8061064 (chr16:53912364)

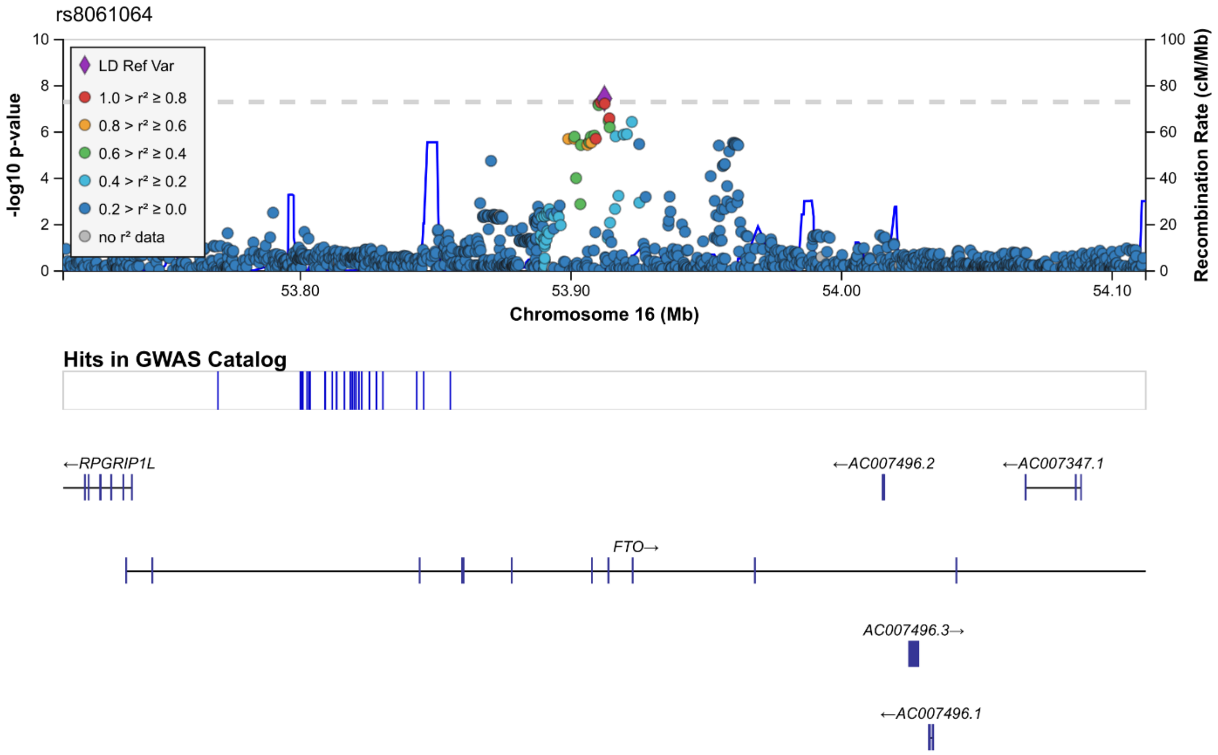

Supplementary Figure 2O: rs12140813 (chr1:227776827)

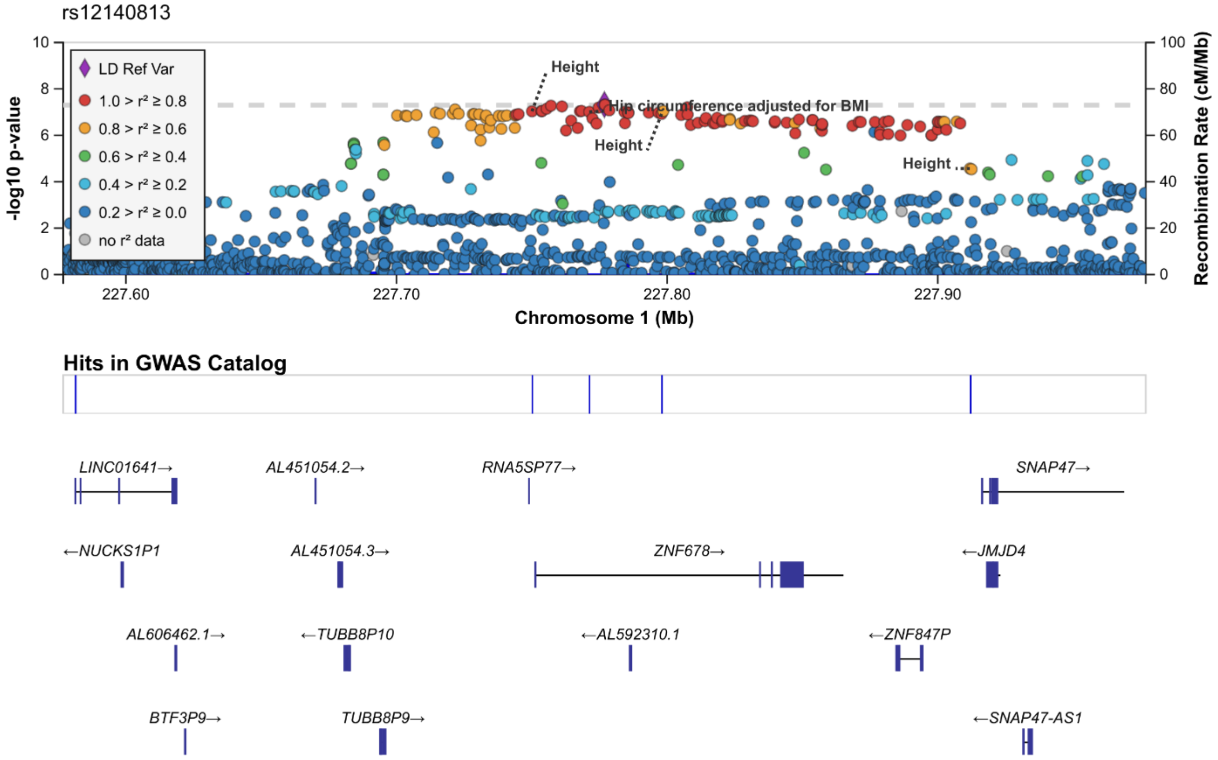

Supplementary Figure 3: Plot of Mendelian randomisation analysis of depression SNPs on low grip strength (EWGSOP) in females only

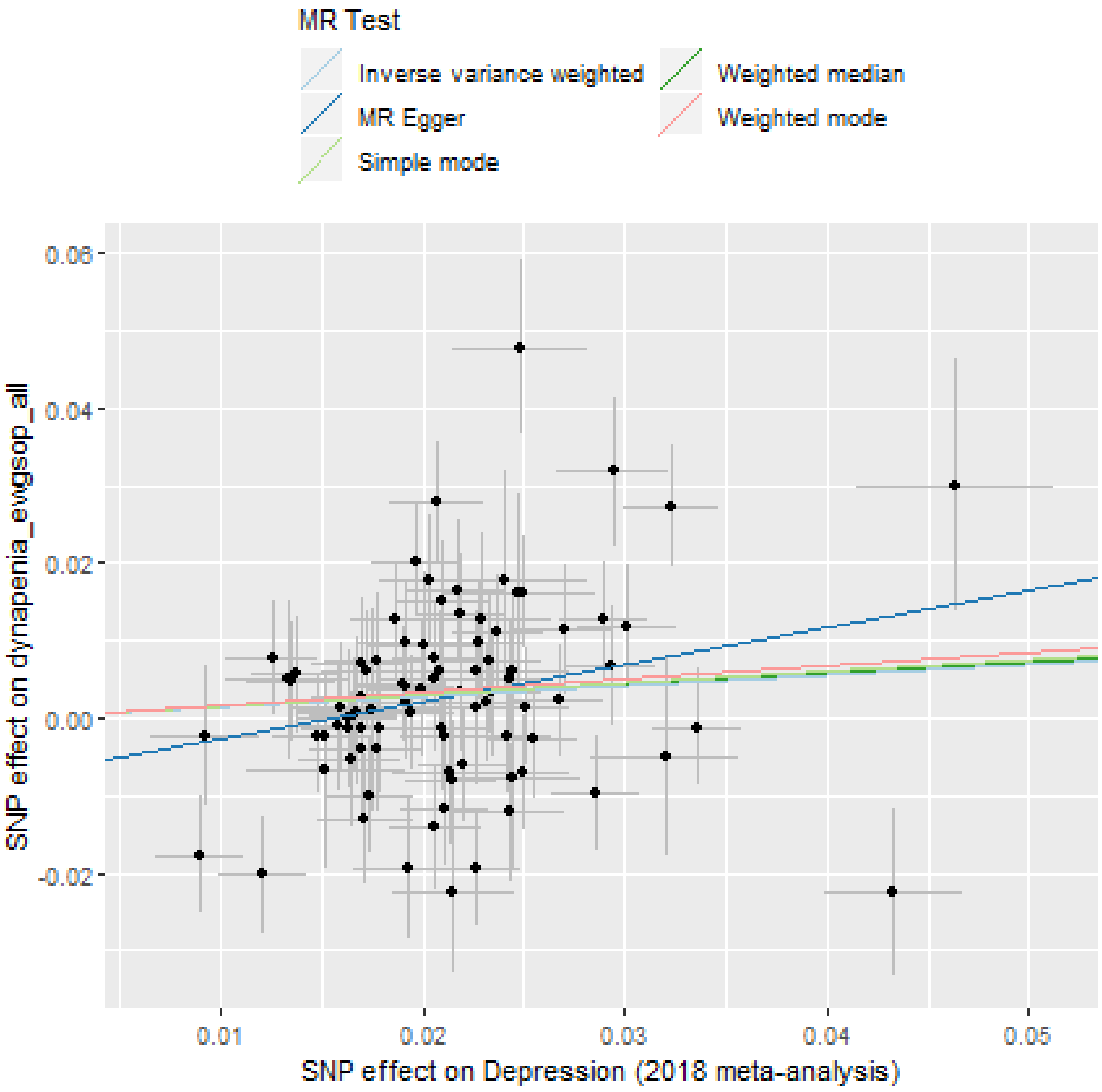

Scatter plot of estimated SNP effects on low grip strength (Dynapenia) European Working Group on Sarcopenia definition against estimated SNP effects on Depression; GWAS meta-analysis N = 132,443 female participants (biologically independent samples) with n=33,548 cases of EWGSOP defined low grip strength; Data points indicate SNP effect on each trait +/- Standard Error

Supplementary Table 1: Summary of cohorts included in the meta-analysis

Study	Number of Females aged >= 60 meeting EWGSOPv.1 low grip criteria (<20kg)	Number of Males aged >= 60 meeting EWGSOPv.1 low grip criteria (<30kg)	Number of Females aged >= 60 meeting FNIH low grip criteria (<16kg)	Number of Males aged >= 60 meeting FNIH low grip criteria (<26kg)	Number of Females Total	Number of Males Total	Number - total
ARIC	650	289	217	134	2,025	1,630	3,655
BASE-II	55	19	5	4	779	752	1,531
BPROOF	328	145	137	64	1,244	1,275	2,519
CHS	601	201	231	88	1,855	1,206	3,061
EPIC-Norfolk	926	503	326	219	3,962	3,546	7,508
FHS	400	142	153	60	1,461	1,245	2,706
HRS	2,380	1,244	1,089	665	6,164	4,650	10,814
InCHIANTI	162	75	90	48	458	361	819
LASA I	121	77	43	37	249	255	504
LASA II	192	85	68	36	632	589	1,221
Long Life Family Study	824	586	446	397	1,788	1,571	3,359
MrOS Gothenburg	0	35	0	10	0	941	941
MrOS Malmo	0	29	0	12	0	891	891
ROSMAP 1	661	198	346	129	1,096	473	1,569
ROSMAP 2	197	48	108	32	266	95	361
Rotterdam Study I	511	234	253	121	853	587	1,440
Rotterdam Study II	273	137	121	58	684	565	1,249
Rotterdam Study III	146	86	41	36	844	640	1,484
SHIP	96	35	43	8	523	513	1,036
TSHA	844	447	525	316	1,218	882	2,100
UK Biobank	24,229	8,807	8,966	3,941	105,597	94,968	200,565
WLS	993	585	393	319	3,770	3,420	7,190
**Total**	**34,589**	**14,007**	**13,601**	**6,734**	**135,468**	**121,055**	**256,523**
	EWGSOP total=	**48,596**	***FNIH total=***	**20,335**			

Supplementary Table 2: Genomic risk loci associated with low grip strength EWGSOP definition, Female only

			EWGSOP Female					EWGSOP All		EWGSOP Male		
**RSID**	**Chr**	**Position**	**P-value**	**EA**	**OA**	**EAF**	**Effect**	**P-value**	**Effect**	**P-value**	**Effect**	**Nearest gene***
rs34415150	6	32560477	1.2E-16	G	A	0.18	0.1001	4.42E-17	0.0833	1.02E-03	0.0582	HLA-DRB1
rs143384	20	34025756	5.87E-13	A	G	0.59	0.0657	4.47E-13	0.0545	2.45E-03	0.0401	GDF5
rs201754	13	51078446	4.43E-10	C	T	0.22	0.0668	3.30E-10	0.0554	3.97E-02	0.0321	DLEU1
rs4764133	12	15064363	3.9E-09	T	C	0.38	0.054	1.92E-09	0.0454	2.42E-02	0.03	ERP27
rs2899611	15	58327347	1.03E-08	G	T	0.51	0.0515	6.01E-09	0.0431	5.33E-03	0.0363	ALDH1A2
rs11236213	11	74394369	2.72E-08	G	A	0.69	0.054	3.01E-10	0.0504	3.13E-04	0.0508	RN7SKP297
rs7185040	16	2145787	3.36E-08	C	A	0.18	0.0642	5.57E-08	0.052	3.91E-02	0.0348	PKD1
rs550258	18	46860643	4.81E-08	T	C	0.35	0.0511	8.51E-10	0.0473	2.23E-03	0.0414	DYM

EA = Effect allele = dynapenia likelihood-increasing allele (flipped if necessary for all related columns)

OA = Other allele

EAF = Effect allele frequency

Effect = beta for association with dynapenia from METAL meta-analysis

Nearest gene = FUMA-annotated gene nearest to lead variant

Supplementary Table 3: Genomic risk loci associated with low grip strength EWGSOP definition, Male only

			EWGSOP Male					EWGSOP All		EWGSOP Female		
**RSID**	**Chr**	**Position**	**P-value**	**EA**	**OA**	**EAF**	**Effect**	**P-value**	**Effect**	**P-value**	**Effect**	**Nearest gene***
rs774787160	21	41996684	1.39E-08	A	ACACATCCAAAAG	0.73	0.0983	2.99E-03	0.0275	9.27E-01	-0.001	DSCAM
rs145933237	3	31188133	2.05E-08	C	G	0.02	0.2688	2.04E-06	0.1344	1.06E-02	0.0898	hsa-mir-466
rs35225200	4	1.03E+08	2.91E-08	C	A	0.08	0.1289	1.67E-10	0.0859	8.46E-05	0.0647	SLC39A8

EA = Effect allele = dynapenia likelihood-increasing allele (flipped if necessary for all related columns)

OA = Other allele

EAF = Effect allele frequency

Effect = beta for association with dynapenia from METAL meta-analysis

Nearest gene = FUMA-annotated gene nearest to lead variant

Supplementary Table 4: Genomic risk loci associated with low grip strength FNIH definition

RSID	Chr	Position	EA	OA	EAF	Effect	P-value	Nearest gene*	GTEx gene expression increased ‡	GTEx gene expression decreased
rs34415150	6	32560477	G	A	0.18	0.1158	5.61E-16	HLA-DRB1	HLA-DQA2; HLA-DRB6; HLA-DQB2; HLA-DOB	HLA-DQA1; HLA-DRB1; HLA-DQB1; HLA-DQB1-AS1
rs3771501	2	70717653	A	G	0.47	0.0697	5.04E-11	TGFA	TGFA (Ts)†	TGFA (Bcor, Bcau, Bhyp, Bacc)†
rs1403785912*	9	4284961	AT	A	0.52	0.0765	4.32E-09	GLIS3	RP11-358M14.2*	
rs12456780	18	46947541	T	A	0.33	0.0633	1.29E-08	DYM		DYM
rs191252760	4	61454905	G	A	0.02	0.1863	3.17E-08	AC095061.1		ADGRL3

† GTEx v8 differential expression by tissue – Ts = Testis; Bcor = Brain – Cortex; Bcau = Brain - Caudate (basal ganglia); Bhyp = Brain – Hypothalamus; Bacc = Brain – Anterior cingulate cortex; Asub = Adipose – subcutaneous; Wb = Whole blood; Esom = Esophagus – mucosa; Skse = Skin sun exposed lower leg; Snse = Skin non-sun exposed lower leg; Thy = Thyroid; Adips = Adipose – Subcutaneous; Adipv = Adipose - Visceral (Omentum); Esog = Esophagus - Gastroesophageal Junction; Haa = Heart - Atrial Appendage; Hlv = Heart - Left Ventricle; Liv = Liver.

* SNP not found in GTEx v8 - rs3934283 used as proxy (r2=0.995, FNIH low grip p-value=3.02*10-6)

EA = Effect allele = dynapenia likelihood-increasing allele (flipped if necessary for all related columns)

OA = Other allele

EAF = Effect allele frequency

Effect = beta for association with dynapenia from METAL meta-analysis

Nearest gene = FUMA-annotated gene nearest to lead variant

Supplementary Table 5: Genetic correlations with low grip strength (EWGSOP)

Phenotype	rg	se	p	h2_obs	h2_obs_se	h2_int	h2_int_se	gcov_int	gcov_int_se	Reference
Osteoarthritis	0.2972	0.0626	2.0E-06	0.0635	0.0085	1.0099	0.0064	0.0413	0.0048	Zengini 2018 (PMID: 29559693)
Coronary Artery Disease	0.1948	0.0301	9.1E-11	0.057	0.0031	0.9934	0.015	0.0158	0.0059	van der Haarst 2018 (PMID: 29212778)
Type-2 Diabetes	0.1582	0.0307	2.7E-07	0.1952	0.0095	1.0782	0.0232	1.80E-02	0.0067	Mahajan 2018 (PMID: 30297969)
Alzheimer's Disease	0.157	0.0787	4.6E-02	0.0079	0.0047	1.0526	0.058	0.0093	0.0048	Jansen 2019 (PMID 30617256)
Rheumatoid Arthritis	0.1267	0.0413	2.1E-03	0.1473	0.0247	0.943	0.0114	0.0053	0.0044	Okada 2014 (PMID: 24390342)
Ischemic Stroke	0.1078	0.0546	4.8E-02	0.0128	0.0014	1.0349	0.0073	0.0093	0.0053	Malik 2018 (PMID: 29531354)
Breast Cancer	0.0453	0.0315	1.5E-01	0.1358	0.0114	1.0917	0.02	0.0114	0.0059	Michailidou 2017 (PMID: 29059683)
Osteoporotic Fracture Risk	0.0164	0.0514	7.5E-01	0.0216	0.0024	0.9877	0.0065	0.0109	0.0047	Trajanoska 2018 (PMID: 30158200)
Chronic Kidney Disease	0.0042	0.0491	9.3E-01	0.167	0.0661	0.9522	0.014	0.0044	0.0049	Pattaro 2016 (PMID: 26831199)
Prostate Cancer	0.0037	0.0484	9.4E-01	0.0575	0.0069	1.071	0.0226	0.0049	0.0053	Schumacher 2018 (PMID: 29892016)
Waist:hip Ratio (adj. BMI)	0.1298	0.0274	2.3E-06	0.1162	0.0056	1.1379	0.0267	0.0384	0.0071	Pulit 2019 (PMID: 30239722)
Body Mass Index	0.0908	0.0249	2.7E-04	0.2091	0.0064	1.031	0.0257	0.0075	0.0081	Yengo 2018 (PMID: 30124842)
Appendicular lean muscle mass	-0.265	0.0582	5.3E-06	0.1804	0.0205	0.995	0.0073	-0.0111	0.0057	Zillikens 2017 (PMID: 28724990)
Whole-body lean muscle mass	-0.3089	0.0607	3.6E-07	0.131	0.0152	0.9993	0.0077	-0.0106	0.006	Zillikens 2017 (PMID: 28724990)
Height	-0.3739	0.0232	2.1E-58	0.4337	0.017	1.6406	0.0918	-0.1278	0.0148	Yengo 2018 (PMID: 30124842)
Grip strength	-0.952	0.0167	0.0E+00	0.1355	0.0047	1.0718	0.017	-0.4084	0.0095	Analysis in UK Biobank Europeans

LD Score Regression methods using published GWAS summary statistics (https://github.com/bulik/ldsc)

Phenotype = GWAS for genetic correlation with dynapenia

rg = genetic correlation, se = standard error of rg, p = p-value for rg

h2_obs, h2_obs_se = observed scale h2 for trait 2 and standard error

h2_int, h2_int_se = single-trait LD Score regression intercept for trait 2 and standard error

gcov_int, gcov_int_se = cross-trait LD Score regression intercept and standard error.

Supplementary Table 6: ICD-10 and UK Biobank self-reported codes used in sensitivity analysis

Condition	UK Biobank self-reported fields	ICD-10 codes	OPCS codes	N
Osteoarthritis	1465	M15.0; M15.1; M15.2; M15.9; M16.0; M16.1; M17.0; M17.1; M18.0; M18.1; M19.0	O18*; W40*; W41*; W42*; W37*; W38*; W39*; W46*; W47*; W48*; W93*; W94*; W95*	29,380
Rheumatoid arthritis	1464	M05; M06		3,263
Rhizarthrosis		M18; M180; M181; M182; M183; M184; M185; M189		355
Osteoporosis	1309	M80; M81		6,308
Dupuytren's contracture	1544	M720; M7204	T521; T522; T525; T526; T541; T561; T562	1,455
Any autoimmune condition	1234; 1522; 1428; 1464; 1381; 1313; 1382; 1261; 1456; 1463; 1477; 1453; 11222	D69*; D758; D141; D862; E271; E05; E051; E063; E100; E101; E102; E103; E104; E105; E106; E107; E108; E109; G35; G737; G70*; H30*; K900; K510; K512; K513; K514; K515; K518; K519; L40; L400; L401; L402; L403; L404; L405; L408; L409; L12*; L10*; L511; M023; M0230; M0236; M0239; M028; M0281; M0284; M0285; M0286; M0287; M029; M0290; M0293; M0294; M0295; M0296; M0297; M0299; M06*; M320; M321; M328; M329; M45; M350; M30*; M34*; M352		16,951

Supplementary References

1. The ARIC investigators. The Atherosclerosis Risk in Communities (ARIC) Study: design and objectives. The ARIC investigators. *Am J Epidemiol*. 1989;129(4):687-702. http://www.ncbi.nlm.nih.gov/pubmed/2646917.
2. Chanda P, Huang H, Arking DE, Bader JS. Fast Association Tests for Genes with FAST. Veitia RA, ed. *PLoS One*. 2013;8(7):e68585. doi:10.1371/journal.pone.0068585.
3. Bertram L, Böckenhoff A, Demuth I, et al. Cohort Profile: The Berlin Aging Study II (BASE-II)†. *Int J Epidemiol*. 2014;43(3):703-712. doi:10.1093/ije/dyt018.
4. J.P. van W, R.A. D-R, N.M. van S, et al. Rationale and design of the B-PROOF study, a randomized controlled trial on the effect of supplemental intake of vitamin B12 and folic acid on fracture incidence. *BMC Geriatr*. 2011;11:80. http://ovidsp.ovid.com/ovidweb.cgi?T=JS&PAGE=reference&D=emed10&NEWS=N&AN=22136481.
5. Fried LP, Borhani NO, Enright P, et al. The cardiovascular health study: Design and rationale. *Ann Epidemiol*. 1991;1(3):263-276. doi:10.1016/1047-2797(91)90005-W.
6. DAWBER TR, KANNEL WB. The Framingham Study An Epidemiological Approach to Coronary Heart Disease. *Circulation*. 1966;34(4):553-555. doi:10.1161/01.CIR.34.4.553.
7. Feinleib M, Kannel WB, Garrison RJ, McNamara PM, Castelli WP. The framingham offspring study. Design and preliminary data. *Prev Med (Baltim)*. 1975;4(4):518-525. doi:10.1016/0091-7435(75)90037-7.
8. Splansky GL, Corey D, Yang Q, et al. The Third Generation Cohort of the National Heart, Lung, and Blood Institute’s Framingham Heart Study: Design, Recruitment, and Initial Examination. *Am J Epidemiol*. 2007;165(11):1328-1335. doi:10.1093/aje/kwm021.
9. Fisher GG, Ryan LH. Overview of the health and retirement study and introduction to the special issue. *Work Aging Retire*. 2018;4(1):1-9. doi:10.1093/workar/wax032.
10. Juster FT, Suzman R. An Overview of the Health and Retirement Study. *J Hum Resour*. 1995;30:S7. doi:10.2307/146277.
11. Howie B, Fuchsberger C, Stephens M, Marchini J, Abecasis GR. Fast and accurate genotype imputation in genome-wide association studies through pre-phasing. *Nat Genet*. 2012;44(8):955-959. doi:10.1038/ng.2354.
12. Loh P-R, Tucker G, Bulik-Sullivan BK, et al. Efficient Bayesian mixed-model analysis increases association power in large cohorts. *Nat Genet*. 2015;47(3):284-290. doi:10.1038/ng.3190.
13. Ferrucci L, Bandinelli S, Benvenuti E, et al. Subsystems contributing to the decline in ability to walk: bridging the gap between epidemiology and geriatric practice in the InCHIANTI study. *J Am Geriatr Soc*. 2000;48(12):1618-1625. doi:10.1111/j.1532-5415.2000.tb03873.x.
14. Melzer D, Perry JRB, Hernandez D, et al. A genome-wide association study identifies protein quantitative trait loci (pQTLs). *PLoS Genet*. 2008;4(5):e1000072. doi:10.1371/journal.pgen.1000072.
15. Das S, Forer L, Schönherr S, et al. Next-generation genotype imputation service and methods. *Nat Genet*. 2016;48(10):1284-1287. doi:10.1038/ng.3656.

16. Hoogendijk EO, Deeg DJH, Poppelaars J, et al. The Longitudinal Aging Study Amsterdam: cohort update 2016 and major findings. *Eur J Epidemiol*. 2016;31(9):927-945. doi:10.1007/s10654-016-0192-0.

17. Huisman M, Poppelaars J, van der Horst M, et al. Cohort Profile: The Longitudinal Aging Study Amsterdam. *Int J Epidemiol*. 2011;40(4):868-876. doi:10.1093/ije/dyq219.

18. Lam M, Awasthi S, Watson HJ, et al. RICOPILI: Rapid Imputation for COnsortias PIpeLIne. Schwartz R, ed. *Bioinformatics*. August 2019. doi:10.1093/bioinformatics/btz633.

19. McCarthy S, Das S, Kretzschmar W, et al. A reference panel of 64,976 haplotypes for genotype imputation. *Nat Genet*. 2016;48(10):1279-1283. doi:10.1038/ng.3643.

20. Das S, Forer L, Schönherr S, et al. Next-generation genotype imputation service and methods. *Nat Genet*. 2016;48(10):1284-1287. doi:10.1038/ng.3656.

21. Sebastiani P, Hadley EC, Province M, et al. A family longevity selection score: ranking sibships by their longevity, size, and availability for study. *Am J Epidemiol*. 2009;170(12):1555-1562. doi:10.1093/aje/kwp309.

22. Abecasis GR, Cherny SS, Cookson WO, Cardon LR. GRR: graphical representation of relationship errors. *Bioinformatics*. 2001;17(8):742-743. doi:10.1093/bioinformatics/17.8.742.

23. Price AL, Patterson NJ, Plenge RM, Weinblatt ME, Shadick NA, Reich D. Principal components analysis corrects for stratification in genome-wide association studies. *Nat Genet*. 2006;38(8):904-909. doi:10.1038/ng1847.

24. Heath SC. Markov chain Monte Carlo segregation and linkage analysis for oligogenic models. *Am J Hum Genet*. 1997;61(3):748-760. doi:10.1086/515506.

25. Mellström D, Johnell O, Ljunggren O, et al. Free testosterone is an independent predictor of BMD and prevalent fractures in elderly men: MrOS Sweden. *J Bone Miner Res*. 2006;21(4):529-535. doi:10.1359/jbmr.060110.

26. A. Bennett D, A. Schneider J, Arvanitakis Z, S. Wilson R. Overview and Findings from the Religious Orders Study. *Curr Alzheimer Res*. 2012;9(6):628-645. doi:10.2174/156720512801322573.

27. Ikram MA, Brusselle GGO, Murad SD, et al. The Rotterdam Study: 2018 update on objectives, design and main results. *Eur J Epidemiol*. 2017;32(9):807-850. doi:10.1007/s10654-017-0321-4.

28. Volzke H, Alte D, Schmidt CO, et al. Cohort Profile: The Study of Health in Pomerania. *Int J Epidemiol*. 2011;40(2):294-307. doi:10.1093/ije/dyp394.

29. Bycroft C, Freeman C, Petkova D, et al. The UK Biobank resource with deep phenotyping and genomic data. *Nature*. 2018;562(7726):203-209. doi:10.1038/s41586-018-0579-z.

30. Thompson WD, Tyrrell J, Borges M-C, et al. Association of maternal circulating 25(OH)D and calcium with birth weight: A mendelian randomisation analysis. *PLoS Med*. 2019;16(6):e1002828. doi:10.1371/journal.pmed.1002828.

31. Herd P, Carr D, Roan C. Cohort Profile: Wisconsin longitudinal study (WLS). *Int J Epidemiol*. 2014;43(1):34-41. doi:10.1093/ije/dys194.

32. Rylander-Rudqvist T. Quality and Quantity of Saliva DNA Obtained from the Self-administrated Oragene Method--A Pilot Study on the Cohort of Swedish Men. *Cancer*

Epidemiol Biomarkers Prev. 2006;15(9):1742-1745. doi:10.1158/1055-9965.EPI-05-0706.

33. Loh PR, Kichaev G, Gazal S, Schoech AP, Price AL. Mixed-model association for biobank-scale datasets. *Nat Genet*. 2018;50(7):906-908. doi:10.1038/s41588-018-0144-6.

34. Lloyd-Jones LR, Robinson MR, Yang J, Visscher PM. Transformation of Summary Statistics from Linear Mixed Model Association on All-or-None Traits to Odds Ratio. *Genetics*. 2018;208(4):1397-1408. doi:10.1534/genetics.117.300360.

35. Willer CJ, Li Y, Abecasis GR. METAL: fast and efficient meta-analysis of genomewide association scans. *Bioinformatics*. 2010;26(17):2190-2191. doi:10.1093/bioinformatics/btq340.

36. Bulik-Sullivan BK, Loh P-R, Finucane HK, et al. LD Score regression distinguishes confounding from polygenicity in genome-wide association studies. *Nat Genet*. 2015;advance on(3):291-295. doi:10.1038/ng.3211.
