# Supplementary material for: Genome-wide meta-analysis of muscle weakness identifies 15 susceptibility loci in older men and women
Source: Nat Commun. 2021 Jan 28;12:654. doi: 10.1038/s41467-021-20918-w (PMC7844411; doi:10.1038/s41467-021-20918-w)
Supplement: Supplementary file 3 — Reporting Summary [file 41467_2021_20918_MOESM3_ESM.pdf]

nature research

Corresponding author(s): Dr Luke C Pilling (L.Pilling@exeter.ac.uk)

Last updated by author(s): Nov 20, 2020

Reporting Summary

Nature Research wishes to improve the reproducibility of the work that we publish. This form provides structure for consistency and transparency in reporting. For further information on Nature Research policies, see our Editorial Policies and the Editorial Policy Checklist.

Statistics

For all statistical analyses, confirm that the following items are present in the figure legend, table legend, main text, or Methods section.

n/a	Confirmed	
☐	☒	The exact sample size (*n*) for each experimental group/condition, given as a discrete number and unit of measurement
☐	☒	A statement on whether measurements were taken from distinct samples or whether the same sample was measured repeatedly
☐	☒	The statistical test(s) used AND whether they are one- or two-sided<br>*Only common tests should be described solely by name; describe more complex techniques in the Methods section.*
☐	☒	A description of all covariates tested
☐	☒	A description of any assumptions or corrections, such as tests of normality and adjustment for multiple comparisons
☐	☒	A full description of the statistical parameters including central tendency (e.g. means) or other basic estimates (e.g. regression coefficient) AND variation (e.g. standard deviation) or associated estimates of uncertainty (e.g. confidence intervals)
☐	☒	For null hypothesis testing, the test statistic (e.g. *F*, *t*, *r*) with confidence intervals, effect sizes, degrees of freedom and *P* value noted<br>*Give P values as exact values whenever suitable.*
☒	☐	For Bayesian analysis, information on the choice of priors and Markov chain Monte Carlo settings
☒	☐	For hierarchical and complex designs, identification of the appropriate level for tests and full reporting of outcomes
☐	☒	Estimates of effect sizes (e.g. Cohen's *d*, Pearson's *r*), indicating how they were calculated

Our web collection on statistics for biologists contains articles on many of the points above.

Software and code

Policy information about availability of computer code

Data collection	BOLT-LMM (v2.3.2 https://alkesgroup.broadinstitute.org/BOLT-LMM/downloads/). Plink (v1.90 beta 6 https://www.cog-genomics.org/plink2/). METAL (v2011-03-25 http://csg.sph.umich.edu/abecasis/Metal/). LDSC (v1.0.1 https://github.com/bulik/ldsc). MetaXcan (v0.6.11 https://github.com/hakyimlab/MetaXcan). R (v3.5.2). LocusZoom (https://locuszoom.org)
Data analysis	Custom shell and R scripts are used for running published programs and packages (referenced in the manuscript) on the associated GitHub repository (https://github.com/pasted/gw_meta_analysis_low_muscle_strength), with additional information available upon request to the authors.

For manuscripts utilizing custom algorithms or software that are central to the research but not yet described in published literature, software must be made available to editors and reviewers. We strongly encourage code deposition in a community repository (e.g. GitHub). See the Nature Research guidelines for submitting code & software for further information.

Data

Policy information about availability of data

All manuscripts must include a data availability statement. This statement should provide the following information, where applicable:

- Accession codes, unique identifiers, or web links for publicly available datasets
- A list of figures that have associated raw data
- A description of any restrictions on data availability

The GWAS summary statistics and supporting information on low grip strength in older people are available on the Musculoskeletal Knowledge Portal (www.mskkp.org) and the GWAS catalog (www.ebi.ac.uk/gwas accession numbers GCST90007526, GCST90007527, GCST90007528, GCST90007529, GCST90007530 and GCST90007531). The International Mouse Phenotyping Consortium database is located (https://www.mousephenotype.org/). eQTL data is available from (https://gtexportal.org/). Catalogue of GWAS associations is available (https://www.ebi.ac.uk/gwas/). All relevant additional data is available on request from the

authors. Information on the 22 individual cohorts is included in the Supplementary Information file.

Field-specific reporting

Please select the one below that is the best fit for your research. If you are not sure, read the appropriate sections before making your selection.

☒ Life sciences ☐ Behavioural & social sciences ☐ Ecological, evolutionary & environmental sciences

For a reference copy of the document with all sections, see nature.com/documents/nr-reporting-summary-flat.pdf

Life sciences study design

All studies must disclose on these points even when the disclosure is negative.

Sample size	The meta-analysis comprised 256,523 individuals of European descent aged 60 years or older at assessment from 22 independent cohorts with maximum hand grip strength recorded and genetics data available - including the UK Biobank, the US Health and Retirement Study (HRS), the Framingham Heart Study (FHS), and others.
Data exclusions	Participants were excluded if they were aged <60 years at time of hand grip assessment, as the pre-established aim of the study is to investigate low grip strength (weakness) in older people. Participants were excluded if missing any outcome or genetic data.
Replication	A 22-cohort meta-analysis was performed, including many sensitivity analyses. No separate "replication" was therefore sought.
Randomization	This genetic study adjusted for relevant covariates in the primary analysis (age, sex and technical covariates), and secondary analysis explored other relevant potential anthropometric and disease confounders.
Blinding	Not relevant because the study is not experimental.

Reporting for specific materials, systems and methods

We require information from authors about some types of materials, experimental systems and methods used in many studies. Here, indicate whether each material, system or method listed is relevant to your study. If you are not sure if a list item applies to your research, read the appropriate section before selecting a response.

Materials & experimental systems

n/a	Involved in the study	
☒	☐	Antibodies
☒	☐	Eukaryotic cell lines
☒	☐	Palaeontology and archaeology
☒	☐	Animals and other organisms
☐	☒	Human research participants
☒	☐	Clinical data
☒	☐	Dual use research of concern

Methods

n/a	Involved in the study	
☒	☐	ChIP-seq
☒	☐	Flow cytometry
☒	☐	MRI-based neuroimaging

Human research participants

Policy information about studies involving human research participants

Population characteristics	All included participants (n=256,523) were aged 60 or over at the time of grip strength assessment, where n=135,468 were female. All participants were genotyped using microarray technology. Please see Supplementary Information file for individual cohort details. Additional analysis in UK Biobank including stratification by diagnosis of the following diseases: osteoarthritis, Rheumatoid arthritis, osteoporosis, Dupuytrens contracture, and rhizarthrosis.
Recruitment	Individual participants from 22 studies contributed data for this meta-analysis. Studies were recruited to the project from the CHARGE consortium (http://www.chargeconsortium.com/) longevity and aging working group, and the GEFOS consortium (http://www.gefos.org/). To participate in the analysis, studies were required to have measured hand grip strength (by dynamometer), all relevant covariates and genetic data. We created an analysis plan with key collaborators which was sent to all studies to perform GWAS in their cohorts, and meta-analysis was performed centrally. Individuals were recruited to e.g. the UK Biobank by postal invitation. Though this will have introduced a healthy volunteer bias at baseline, studies have shown that results are still generalizable to the population (doi:10.1093/aje/kwx246).
Ethics oversight	Individual studies have ethical approval for this study, such as the UK Biobank study where approval was obtained from the North West Multi-Centre Research Ethics Committee. Please see Supplementary Information file for individual cohort details.

Note that full information on the approval of the study protocol must also be provided in the manuscript.
