# Supplementary material for: Genome-wide meta-analysis of muscle weakness identifies 15 susceptibility loci in older men and women
Source: Nat Commun. 2021 Jan 28;12:654. doi: 10.1038/s41467-021-20918-w (PMC7844411; doi:10.1038/s41467-021-20918-w)
Supplement: Supplementary file 4 — Description of Additional Supplementary Files [file 41467_2021_20918_MOESM4_ESM.pdf]

Description of Additional Supplementary Files

Supplementary Data 1 (EWGSOP loci metal results)

Supplementary Data 2 (GWAS catalog summary)

Supplementary Data 3 (GWAS catalog full results)

Supplementary Data 4 (Linear vs. low grip)

Supplementary Data 5 (Mitochondrial variant analysis)

Supplementary Data 6 (eQTL GTEx.v8)

Supplementary Data 7 (GO processes)

Supplementary Data 8 (MetaXcan results)

Supplementary Data 9 (MetaXcan with mouse annotation)

Supplementary Data 10 (LDSC-SEG)

Supplementary Data 11 (TwoSampleMR results)

Supplementary Data 12 (TwoSampleMR results - females)

Supplementary Data 13 (TwoSampleMR results - males)

Supplementary Data 14 (UKB sensitivity analysis)
